# E2FA and E2FB transcription factors coordinate cell proliferation with seed maturation

**DOI:** 10.1242/dev.179333

**Published:** 2019-11-26

**Authors:** Tünde Leviczky, Eszter Molnár, Csaba Papdi, Erika Őszi, Gábor V. Horváth, Csaba Vizler, Viktór Nagy, János Pauk, László Bögre, Zoltán Magyar

**Affiliations:** 1 Institute of Plant Biology, Biological Research Centre, H-6726 Szeged, Hungary; 2 Doctoral School in Biology, Faculty of Science and Informatics, University of Szeged, H-6726 Szeged, Hungary; 3 Centre for Systems and Synthetic Biology, Department of Biological Sciences, Royal Holloway University of London, Egham, Surrey TW20 0EX, UK; 4 Institute of Biochemistry, Biological Research Centre, H-6726 Szeged, Hungary; 5 Cereal Research Non-Profit Ltd Co., H-6726 Szeged, Hungary

**Keywords:** E2F-RBR transcriptional regulatory mechanism, Developmental transitions, Cell proliferation, Seed and embryogenesis, *Arabidopsis*

## Abstract

The E2F transcription factors and the RETINOBLASTOMA-RELATED repressor protein are principal regulators coordinating cell proliferation with differentiation, but their role during seed development is little understood. We show that in fully developed *Arabidopsis thaliana* embryos, cell number was not affected either in single or double mutants for the activator-type *E2FA* and *E2FB*. Accordingly, these E2Fs are only partially required for the expression of cell cycle genes. In contrast, the expression of key seed maturation genes *LEAFY COTYLEDON 1*/*2* (*LEC1*/*2*), *ABSCISIC ACID INSENSITIVE 3*, *FUSCA 3* and *WRINKLED 1* is upregulated in the *e2fab* double mutant embryo. In accordance, E2FA directly regulates LEC2, and mutation at the consensus E2F-binding site in the *LEC2* promoter de-represses its activity during the proliferative stage of seed development. In addition, the major seed storage reserve proteins, 12S globulin and 2S albumin, became prematurely accumulated at the proliferating phase of seed development in the *e2fab* double mutant. Our findings reveal a repressor function of the activator E2Fs to restrict the seed maturation programme until the cell proliferation phase is completed.

## Abstract (precis)

**Highlighted Article:** During seed and embryo development the E2FA and E2FB transcription factors coordinate cell proliferation with differentiation and accumulation of seed reserves; however, they are not essential for sustaining cell proliferation.

## INTRODUCTION

In multicellular organisms, development is regulated by coordinating cell proliferation with differentiation. In plants, owing to their sessile lifestyle and largely post-embryonic development, this coordination operates lifelong, from early embryogenesis to post-embryonic organ development. Plants develop through transitions, but how these passages are regulated at the molecular level is not fully understood. The developing seed consists of two major and sequential programmes; the initial morphogenic phase is driven by oriented cell divisions, and is followed by the maturation phase, in which embryonic cells stop proliferating and seed storage reserves accumulate (Holdsworth et al., 2008; Lau et al., 2012; Sun et al., 2010). During the final phase of embryogenesis, desiccation tolerance is acquired and dormancy is established (Devic and Roscoe, 2016). The embryo formation, the accumulation of storage reserves and the establishment of dormancy are all important agronomic traits that define seed quality (Baud et al., 2008).

Morphogenesis during seed development is completed in the early heart stage embryo, when all elements of the body pattern are already laid down (Wendrich and Weijers, 2013). The embryo still continues to grow afterwards, but mostly by cell expansion rather than by cell proliferation (Raz et al., 2001). Seed storage reserves, including fatty acids and proteins, accumulate when cell division is completed (Goldberg et al., 1994). The current view is that the key genetic factors controlling seed maturation are four regulatory genes, including those encoding three related B3 domain transcription factors, ABSCISIC ACID INSENSITIVE 3 (ABI3), FUSCA 3 (FUS3) and LEAFY COTYLEDON 2 (LEC2), collectively named AFL, and the CCAAT-binding transcription factor LEAFY COTYLEDON 1 (LEC1) (Braybrook and Harada, 2008; Carbonero et al., 2017). The exact mechanism behind initiation of the maturation phase through the control of these genes is, however, still not entirely clear.

Cell proliferation is highly regulated during embryo development. In *Arabidopsis*, as in other eukaryotes, cyclin-dependent kinases (CDKs) play essential roles in the regulation of the cell cycle (Gutierrez, 2009). Contrary to animals, *Arabidopsis* embryos can develop in the absence of the evolutionarily conserved CDKA;1, but contain many fewer cells. The primary target for CDKA;1 is the single RETINOBLASTOMA-RELATED (RBR) protein, which was experimentally demonstrated with the rescue of most defects in the *cdka;1* mutant by the *rbr1-2* hypomorph mutant allele (Nowack et al., 2012). As the main RBR-kinase is CDKA;1, it forms a complex with regulatory cyclin subunits, including D-type cyclins (CYCDs). CYCDs have both discrete and overlapping tissue-specific expression patterns in the developing seeds and mutations of the CYCD3 subgroup delay embryo development (Collins et al., 2012). CYCDs bind to retinoblastoma protein (Rb/RBR) through their LxCxE amino acid motif, which leads to the phosphorylation and inactivation of Rb/RBR (Morgan, 2007; Boniotti and Gutierrez, 2001). The canonical role of RBR is to control the cell cycle through the repression of E2F transcription factors (De Veylder et al., 2007; Harashima and Sugimoto, 2016). In *Arabidopsis*, three E2F proteins are capable of forming complexes with RBR (Magyar et al., 2016). Ectopic expression of E2FA or E2FB causes hyper-proliferation, whereas overexpression of E2FC inhibits cell division during post-embryonic development, placing them as activator and repressor type E2Fs, respectively (De Veylder et al., 2002; del Pozo et al., 2006; Magyar et al., 2005, 2012; Sozzani et al., 2006). These three E2Fs require the dimerisation partner protein A (DPA) or B (DPB) for DNA binding (del Pozo et al., 2002, 2006; Magyar et al., 2000). Only E2FB and E2FC, but not E2FA, were found in association with components of the evolutionarily conserved multisubunit DP-Rb-E2F And-MuvB complex (DREAM; Kobayashi et al., 2015; Fischer and DeCaprio, 2015; Sadasivam and DeCaprio, 2013), demonstrating that activator E2FA and E2FB could have different functions (Horvath et al., 2017). Accordingly, E2FA in complex with RBR was shown to maintain the proliferation competence by repressing genes controlling the switch from mitosis to endocycle and cell elongation (Magyar et al., 2012), whereas E2FB was shown to regulate cell cycle in a more canonical way, with RBR repressing the activation of cell cycle genes through the inhibition of E2FB. The function of these E2Fs in the developing embryo has not yet been fully characterised. Mutant embryos with compromised RBR function develop normally, but consist of twice as many cells as the wild type (Gutzat et al., 2011). Cell number in this *rbr* mutant increased from the bent cotyledon embryo stage onward during maturation, suggesting that RBR repression is required for the exit from cell proliferation to set the final cell number in the embryo (Nowack et al., 2012). In addition, *rbr* mutant seedlings ectopically express embryonic genes such as *LEC2* and *ABI3*, indicating that RBR, apart from cell cycle genes, could regulate the expression of seed maturation genes (Gutzat et al., 2012). Whether plant RBR regulates cell proliferation in the developing embryo in association with E2Fs and whether they together control the developmental transitions to seed maturation is not known.

Here, we analysed the function of activator-type E2FA and E2FB in developing *Arabidopsis* seeds and embryos. We found that in the *e2fa-2/e2fb-1* double mutant (*e2fab*; Heyman et al., 2011) cell number was not significantly affected in the fully developed embryos. Accordingly, the activator function of E2FA and E2FB is not crucial for embryonic cell proliferation. In contrast, the expression of the key seed maturation genes *LEC1*/*2*, *ABI3* and *FUS3* was found to be significantly upregulated in *e2fab* embryos. Our findings reveal a repressor function of the so-called activator E2Fs to restrict the seed maturation programme until the cell proliferation phase is completed.

## RESULTS

### The expression patterns of E2FA and E2FB are distinct in developing siliques

**Fig. 1.**
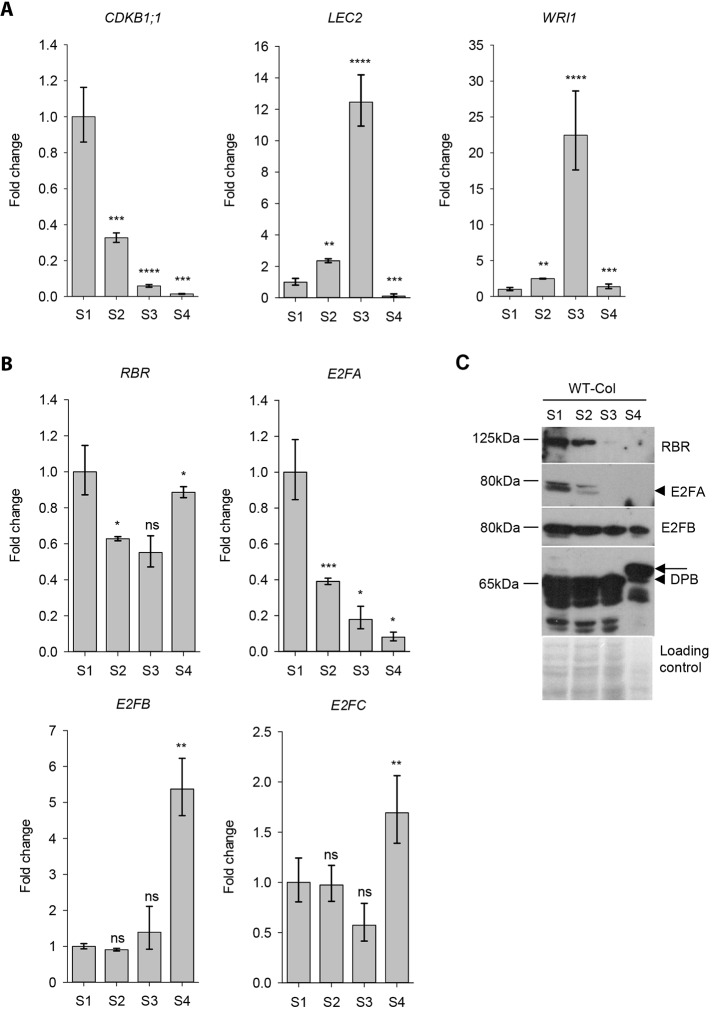
**The expression profiles of E2FA and E2FB are distinct in the developing siliques, but overlap in the proliferation phase.** (A) qRT-PCR analyses of the G2- and M-phase-specific *CDKB1;1* and the seed maturation *LEC2* and *WRI1* genes in the developing siliques of the wild-type (WT) at four silique developmental stages (S1-S4, pictured in Fig. S1). (B) The transcript levels of the three E2Fs, namely *E2FA*, *E2FB* and *E2FC*, and the single *RBR* genes were also analysed in these silique samples by qRT-PCR. Values represent fold-changes normalised to the value of the S1 silique stage (set arbitrarily at 1). Data are mean±s.d., *n*=3 biological repeats. *P≤0.05, **P≤0.01, ***P≤0.001, ****P≤0.0001 (two-tailed, paired *t*-test between consecutive silique stages). ns, non-significant. (C) To follow the accumulation levels of RBR, E2FA, E2FB and DPB proteins in developing siliques (S1-S4) specific antibodies were used in immunoblot assays as indicated. The Ponceau-stained proteins were used as loading control. Arrowheads indicate the corresponding E2FA and DPB proteins; arrow marks a slower migrating form of DPB in S4 silique stage. Molecular weights of the specific proteins are shown on the left.

To investigate the involvement of activator E2Fs in the coordination of cell proliferation and differentiation, we first studied the expression of *E2FA* and *E2FB*. We harvested siliques from *Arabidopsis* wild-type Columbia 0 ecotype (WT) with four different sizes, representing distinct embryo developmental stages (S1-S4; Fig. S1). To monitor the proliferative phase in this experimental system, we studied the expression of *CDKB1;1*, a G2-M phase-specific cell cycle regulatory gene, a known target for activator E2Fs (Vandepoele et al., 2005). *CDKB1;1* was found to express at the highest level in the youngest siliques (S1), this decreased in the second silique sample (S2) and sharply diminished afterwards in the last two silique samples (S3-S4) (Fig. 1A). To monitor the maturation phase, we followed the expression of *LEC2*, and one of its predicted target genes, *WRINKLED 1* (*WRI1*; Focks and Benning, 1998), an APETALA2/ETHYLENE-RESPONSE FACTOR (AP2/ERF)-type transcription factor involved in the regulation of fatty acid synthesis (Fig. 1A). As expected, these were barely detectable during the proliferative phase (S1) and they both showed the highest expressions in the third silique sample (S3), containing long fully grown but green siliques, and both declined in the S4 sample (Fig. 1A). Taken together, cell proliferation was the most active in the youngest siliques (S1 and S2). The maturation phase started when proliferation activity decreased in the transient developmental phase (S2) and peaked in the next sample (S3), and both the cell cycle and maturation genes were hardly detectable in the post-mature seed developmental phase (S4).

To understand the function of E2Fs and RBR during seed development, we followed the transcript levels of the three E2Fs (*E2FA*, *E2FB*, *E2FC*) as well as *RBR*. The repressor type *E2FC* and *RBR* were expressed at nearly constant levels from proliferation to maturation phase of seed development (Fig. 1B). The expression pattern of activator *E2FA* was similar to the cell cycle regulator *CDKB1;1* gene; it was highest in proliferating seeds and gradually decreased afterwards, although not as sharply as the expression of *CDKB1;1* in the post-mitotic S3-S4 siliques, and remained clearly detectable (Fig. 1A,B). *E2FB* was also expressed during the early developmental phases (S1-S2), but unlike *E2FA*, its expression level increased during the maturation phase and it peaked afterwards in the post-mature developmental stage (Fig. 1B). These results are in agreement with the gene expression data in the *Arabidopsis* eFP browser (Fig. S2; Winter et al., 2007), supporting overlapping as well as potentially specific functions for *E2FA* and *E2FB* during silique and seed development.

### E2FA and RBR proteins are abundant in the proliferative phase, whereas E2FB protein is present in post-mitotic and post-mature seeds and siliques

Next we analysed the accumulation of E2FA and E2FB proteins in the developing siliques using specific antibodies in immunoblot assays (Fig. 1C). The E2FA protein accumulation mirrored its transcript level, being highest in the proliferation phase of siliques (S1), decreasing towards the maturation phase in S2 and diminishing in the latest developmental phases (S3-S4; Fig. 1C). RBR is known to be abundant in proliferating tissues during vegetative development (Borghi et al., 2010; Magyar et al., 2012), and indeed the level of RBR was high in the young siliques (S1-S2) but, contrary to its transcript level, RBR protein was hardly detectable in maturing siliques (S3) and further diminished from the post-mature S4 stage, indicating that RBR mRNA and not RBR protein is stored in the dry seeds. In contrast to E2FA and RBR, E2FB accumulated at a constitutive high level throughout seed and silique development, present both in the mitotically active and maturing siliques and interestingly also in the post-mature stage (Fig. 1C). We could not detect DPA in the developing siliques, probably because of its generally low level, but DPB showed a constitutive expression pattern throughout the analysed developmental period, similar to E2FB (Fig. 1C). In the post-mature silique stage (S4), DPB was detected with a slower mobility, indicating a post-translational modification on this protein. The diminished abundance of RBR, but not E2FB, at the post-maturation stage suggests that E2FB may have an RBR-independent function during the establishment of seed dormancy.

### Spatial and temporal regulation of E2FA and E2FB accumulation during embryogenesis

**Fig. 2.**
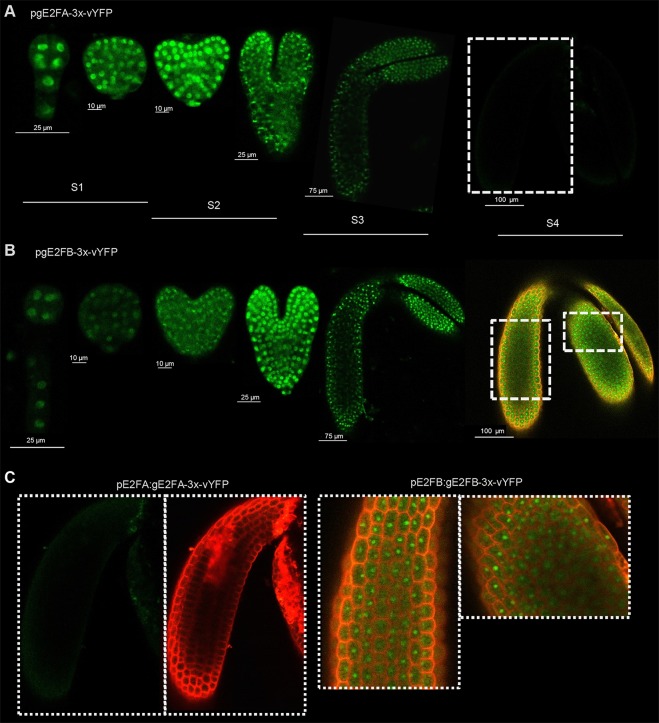
**Spatial and temporal regulation of E2FA and E2FB accumulation during embryogenesis.** (A-C) Representative confocal microscopy images of developing embryos dissected from immature seeds of pgE2FA-3xvYFP (A) and pgE2FB-3xvYFP (B) transgenic lines (O˝szi et al., 2019). White dashed boxes outline the epidermal regions of hypocotyls and cotyledon in the post-mature E2FA-3xvYFP (left side) and E2FB-3xvYFP (right side) embryos, magnified in (C). The vYFP signal is green, the cell wall is counterstained with propidium iodide (PI) (red). The merged images of the YFP and the PI signals are shown for the post-mature E2FB-3xvYFP embryo. Silique stages indicated (S1-S4) correspond to the different embryo developmental phases.

To analyse the spatial and temporal patterns of E2FA, E2FB and RBR proteins specifically in the developing embryos, we used our transgenic *Arabidopsis* lines expressing fluorescent protein-tagged E2FA, E2FB or RBR under the control of their own promoters (pgE2FA-3xvYFP, pgE2FB-3xvYFP, pgRBR-3xCFP; O˝szi et al., 2019). Immature embryos were dissected from transgenic *Arabidopsis* seeds at various developmental stages and fluorescence signals were analysed by confocal laser microscopy (Fig. 2; Fig. S3). Cell proliferation continues during the heart stage, but gradually decreases until the walking-stick embryo stage, when it completely stops (Raz et al., 2001). Both E2FA and E2FB proteins were found to be nuclear, and ubiquitously expressed in every embryonic cell, from the globular to the mitotically quiescent walking-stick embryo stage (Fig. 2). The E2FA-vYFP signal was the brightest till the heart stage, after which it gradually diminished, but remained detectable at all stages except the post-mature phase in S4, whereas the E2FB-vYFP signal was most intense at the torpedo stage, but could be detected in the latest embryo developmental stages (Fig. 2). The RBR-3xCFP was detected from the heart to the walking-stick embryo stage, but it was not present in post-mature embryos (Fig. S3). E2FA-3xvYFP and E2FB-3xvYFP signals were also present in the integuments of young seeds containing proliferating cells (Fig. S4).

Altogether, these results show that both E2FA and E2FB, as well as RBR proteins, are present in the developing embryo both in proliferating and in post-mitotic embryonic cells, though at a different abundance. Accordingly, E2FA and E2FB have the potential to participate in the establishment of quiescence in association with RBR, until the embryo reaches its final size at the S3 stage.

### In the *e2fab* double mutant the expression of cell cycle genes is compromised during the early developmental stage, but it becomes de-repressed later during maturation

**Fig. 3.**
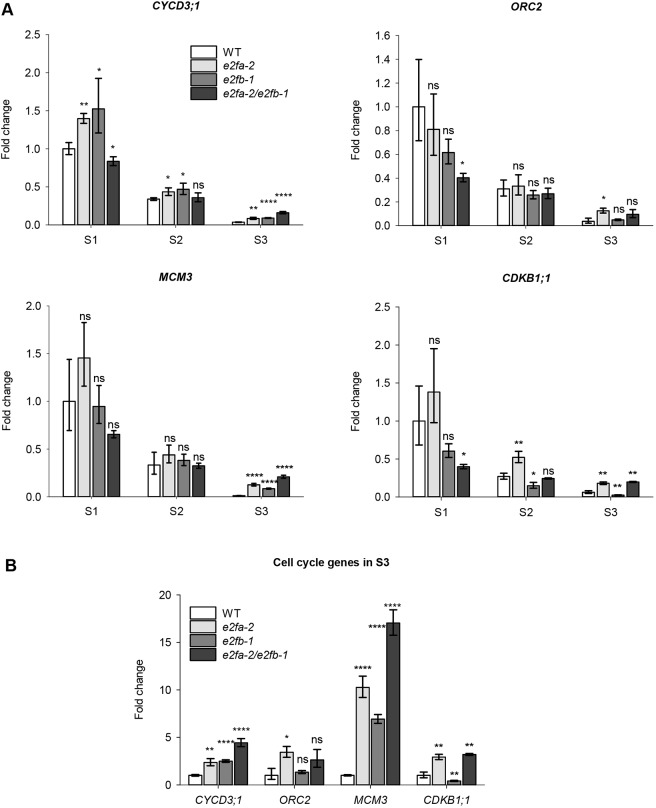
**E2FA and E2FB could function either as activators or repressors on cell cycle genes depending on the developmental stage of siliques and seeds.** (A) Comparison of the *CYCD3;1*, *ORC2*, *MCM3*, and *CDKB1;1* transcript levels in developing siliques of wild-type (WT) and the *e2fa-2*, *e2fb-1* single and *e2fa-2/e2fb-1* double mutants, respectively, at three silique developmental stages (S1-S3). Values represent fold changes normalised to the value of the WT at the S1 silique stage (set arbitrarily at 1). (B) The expression levels of cell cycle genes in the S3 maturation phase are compared between the *e2f* mutants and the control WT. The values represent fold change normalised to the value of the relevant gene from the WT at the S3 silique stage (set arbitrarily at 1). Data are mean±s.d., *n*=3 biological repeats. *P≤0.05, **P≤0.01, ****P≤0.0001 (two-tailed, paired *t*-test between the corresponding mutant and the WT, at a given silique stage). ns, non-significant.

To examine whether E2FA and E2FB are required for the expression of cell cycle genes, we collected siliques at three developmental phases of *e2fa-2* (Berckmans et al., 2011b) and *e2fb-1* (Berckmans et al., 2011a; Horvath et al., 2017) single mutants, as well as the *e2fab* double mutant (Heyman et al., 2011). It has previously been shown that these mutant lines do not express the corresponding full size transcripts and proteins (Berckmans et al., 2011a,b; Horvath et al., 2017; Kobayashi et al., 2015; Figs S9, S10 and S11). We followed the expression of the G1-to-S phase regulatory *CYCD3;1*, the S-phase linked *ORIGIN RECOGNITION COMPLEX 2* (*ORC2*), the *MINICHROMOSOME MAINTENANCE 3* (*MCM3*) and the G2-to-M phase-specific *CDKB1;1* E2F target genes using qRT-PCR (Fig. 3). In the WT siliques, all these cell cycle genes showed a generally similar pattern: highest expression in the first silique sample, representing the proliferation phase, declined levels in the following one, and the lowest during the maturation phase (Fig. 3A). Surprisingly, the expression of these cell cycle genes during the proliferative S1 stage was hardly affected in the single mutant and just lowered in the *e2fab* double mutant, but only marginally in the case of *MCM3* and *CYCD3;1*, suggesting that these activator E2Fs are only partially required for their expressions.

Cell cycle genes almost completely diminished in the maturing siliques of the WT. To evaluate the effect of *e2fa-2* and *e2fb-1* mutations on their expression, we replotted the normalised data representing the S3 stage (Fig. 3B). All these cell cycle genes were upregulated in the *e2fa-2* mutant, whereas only the expression of *CYCD3;1* and *MCM3* was elevated in *the e2fb-1* mutant. These two further increased in the *e2fab* double mutant, suggesting that activator E2Fs act independently as repressors on them. In contrast, *CDKB1;1* expression diminished in the *e2fb-1* mutant, whereas it became elevated in the *e2fab* double to the same level as in the *e2fa-2* single, suggesting that these E2Fs oppositely regulate *CDKB1;1* expression. These results show that the E2FA and E2FB activator-type transcription factors can act as repressors during the maturation phase of seed development.

### E2FA and E2FB are dispensable for embryonic cell proliferation

**Fig. 4.**
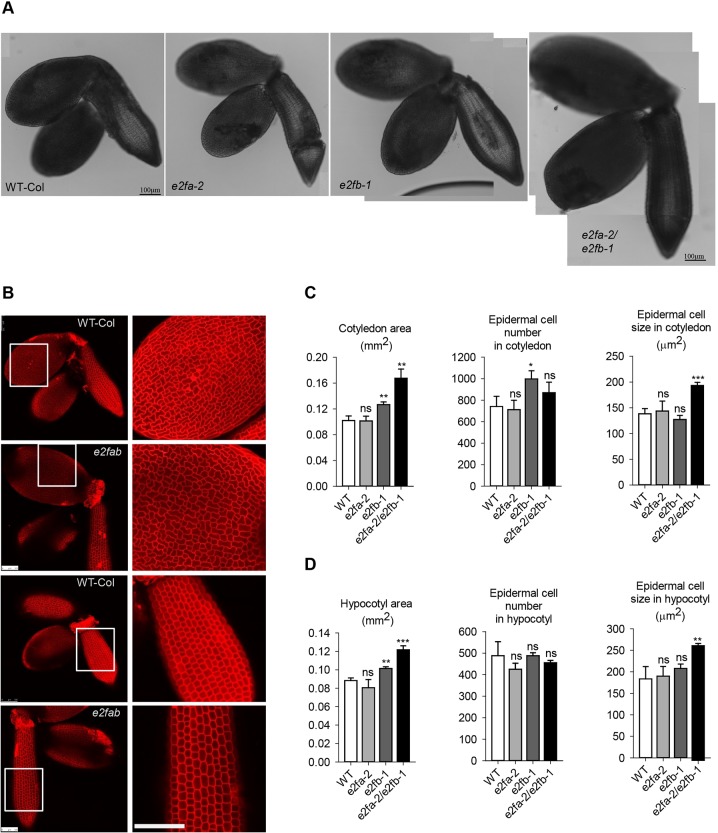
**E2FA and E2FB are dispensable for embryonic cell proliferation.** (A) Representative confocal images of mature embryos from wild type (WT) and *e2fa-2*, *e2fb-1* single and *e2fa-2/e2fb-1* double mutants dissected from mature dry seeds. (B) Confocal images of propidium iodide (PI)-stained WT-Col and *e2fa-2/e2fb-1* double mutant embryos (additional images are shown in Fig. S5). White boxes outline epidermal regions in cotyledons and hypocotyls of WT and *e2fa-2/e2fb-1* double mutants, enlarged on the right to show epidermal cell sizes. Scale bars: 100 µm. (C,D) The entire cotyledon (C) and hypocotyl (D) area of mature dried embryos was measured. Data are mean±s.d., *n*=3 biological repeats, *N*=10 samples in each. Cell size and cell number were calculated using ImageJ. Sample size *N*≥200 cells/image (*n*=4 biological repeats). *P≤0.05, **P≤0.01, ***P≤0.001 (two-tailed, paired *t*-test between the corresponding mutant and the WT). ns, non-significant.

Previous results have confirmed that cell number in the developing *Arabidopsis* embryo is regulated at the level and activity of RBR, which acts on E2Fs (Gutzat et al., 2011; Nowack et al., 2012). To analyse the role of activator E2Fs, we isolated embryos from fully mature seeds of WT, single and double loss-of-function *e2fa-2* and *e2fb-1* mutants and determined the size of embryonic cotyledons and hypocotyl and their constituent cells under confocal laser microscopy after propidium iodide (PI) staining (Fig. 4; Fig. S5). The *e2fa* mutant embryo looked normal, whereas the *e2fb* was slightly larger than the WT (increased by 1.2-fold), containing more but slightly smaller cells (Fig. 4A,C). The double *e2fab* mutant embryos were significantly larger, with enlarged cotyledons and hypocotyl (Fig. 4A,B). However, the number of cells in these *e2fab* mutants was calculated to be comparable with the WT control, whereas the cell size was considerably increased in comparison with WT, both in the cotyledon and in the hypocotyl epidermal tissue (Fig. 4C,D). We also observed that *e2fab* mutant plants produced shorter siliques containing fewer, but bigger and heavier, seeds than the WT (Table 1). The short silique was because of reduced fertility, as indicated by missing rather than aborted seeds in the silique in the *e2fab* double mutant (Fig. S6). Accordingly, the yield of the double *e2fab* mutant plants was behind WT (decreased by ∼30%), whereas seed weight increased by 36%, indicating a negative correlation between total seed yield and average seed weight (Table 1). The large seed and embryo phenotype in this mutant could be the consequence of the allocation of extra resources to the few seeds produced (Venable, 1992; Ohto et al., 2005). Nevertheless, *e2fab* mutant embryos, in the absence of activator E2FA and E2FB functions, are larger than the WT control, although the total number of cells is not modified, supporting the view that the activator function of these E2Fs is not essential for cell proliferation during embryogenesis.

**Table 1.**
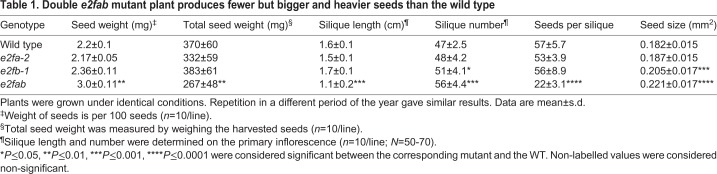
**Double** ***e2fab*** **mutant plant produces fewer but bigger and heavier seeds than the wild type**

### The AFL class of maturation genes are repressed by E2FA and E2FB

**Fig. 5.**
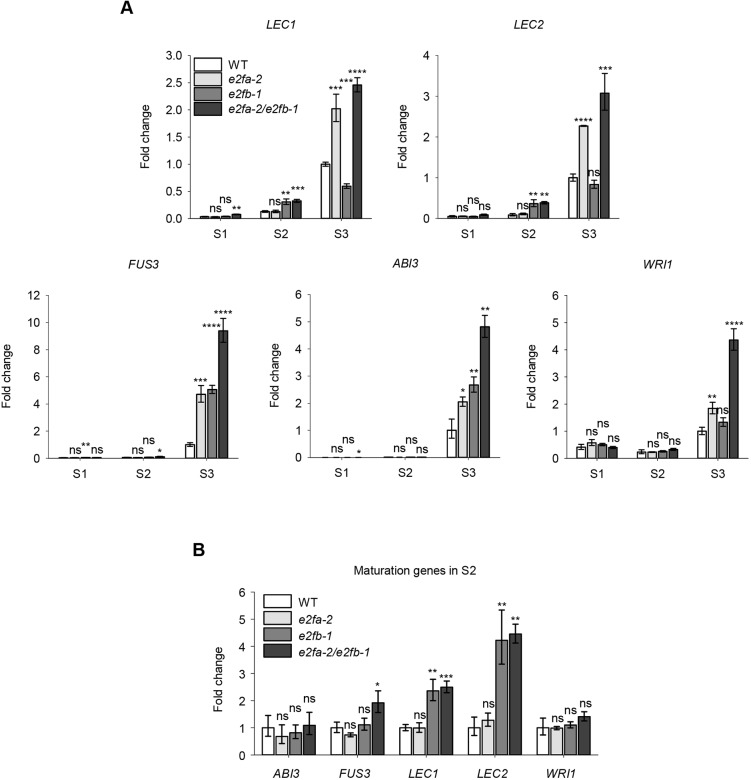
**Activator E2Fs repress key maturation genes in developing siliques and seeds.** (A) Comparison of the *LEC1*, *LEC2*, *FUS3*, *ABI3* and *WRI1* transcript levels in developing siliques of the control (WT) and the *e2fa-2*, *e2fb-1* and *e2fa-2/e2fb-1* single and double mutants, respectively, at three silique developmental stages (S1-S3). Values represent fold changes normalised to the value of the WT at the S3 silique stage (set arbitrarily at 1). (B) The expression levels of maturation genes in the transition S2 phase in the *e2f* mutants and WT. Values represent fold change normalised to the value of the relevant gene from WT at S2 (set arbitrarily at 1). Data are mean±s.d., *n*=3 biological repeats. *P≤0.05, **P≤0.01, ***P≤0.001, ****P≤0.0001 (two-tailed, paired *t*-test between the corresponding mutant and the WT, at a given silique stage). ns, non-significant.

It has previously been shown that seed maturation genes *LEC2* and *ABI3* were upregulated in *Arabidopsis* seedlings, whereas the RBR level was reduced by co-silencing (csRBR; Gutzat et al., 2011). The *LEC2* gene is a putative E2F target, as it contains a consensus E2F-binding site in its promoter, although RBR could not be shown to directly bind to *LEC2*, but only to the *ABI3* promoter (Gutzat et al., 2011). To investigate the role of activator E2Fs, we followed the expression of *LEC2*, *LEC1*, *FUS3* and *ABI3*, as well as *WRI1*, in developing siliques of single and double *e2fa-2* and *e2fb-1* mutants (Fig. 5). As expected, in WT the maturation genes were hardly detectable in the proliferating siliques (S1), they increased afterwards (S2), and peaked during maturation (S3: Fig. 5). The expression of all these maturation genes was upregulated in the *e2fa-2* and partly in the *e2fb-1* mutants during S3 phase. For *LEC1* and *LEC2* this solely depended on *e2fa-1*, whereas for *FUS3*, *ABI3* and to some extent for *WRI1*, this depended on both *e2fa-2* and *e2fb-1* (Fig. 5A). In contrast to the S3 phase, the *LEC1* and *LEC2* transcripts became prematurely upregulated during the S2 phase only in the *e2fb-1* mutant (Fig. 5B). These data indicate that both E2FA and E2FB could repress the *LEC1*/*2* genes, but in different seed developmental stages.

### The expression of *LEC2* and *WRI1* is regulated by E2Fs during silique development

**Fig. 6.**
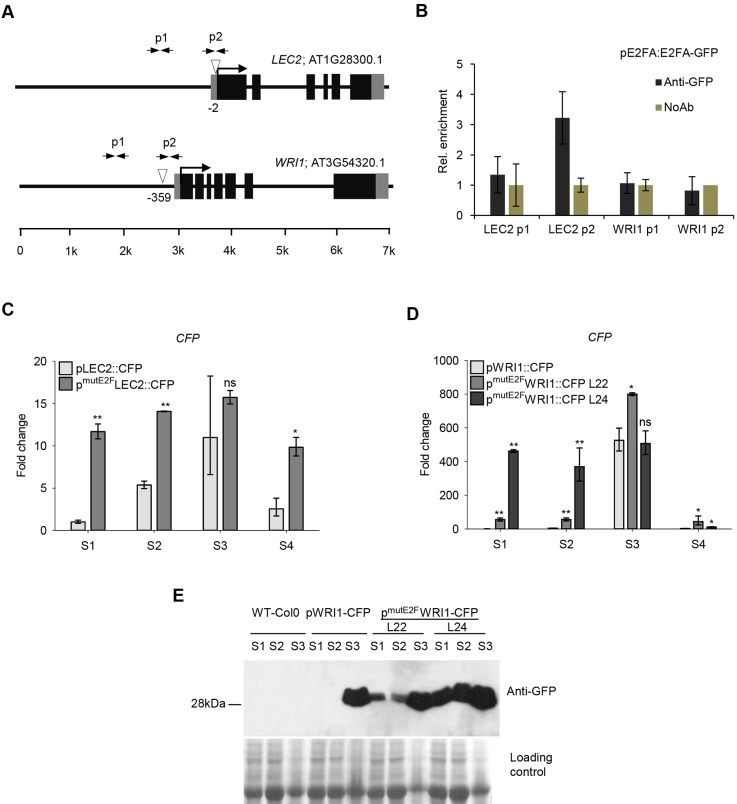
**E2Fs could regulate the temporal control of *LEC2* and *WRI1* genes during silique development.** (A) Schematic of the LEC2 and WRI1 promoters; arrows labelled p1 and p2 indicate the position of the primer pairs used for qPCR analysis. The position of the canonical E2F elements (white arrowheads) and their distance from the start codon (ATG) are depicted. (B) ChIP followed by qRT-PCR was carried out on chromatin isolated from developing green siliques (6-10 DAP) of the pgE2FA-GFP transgenic line using a polyclonal anti-rabbit GFP antibody (Ab). The graph shows the results of a representative experiment with three biological replicates. Non-parametric Mann–Whitney *U*-test was used for statistical analysis between values of Ab and NoAb samples (*P*<0.05). The labels p1 and p2 on the *x*-axis refer to the regions indicated in A. (C,D) The expression levels of reporter LEC2 (C) and WRI1 (D) constructs either under the control of the intact (pLEC2::CFP, pWRI1::CFP, respectively) or the E2F-binding-site-mutant promoter version (p^mutE2F^LEC2::CFP, p^mutE2F^WRI::CFP, respectively) were determined by qRT-PCR in the developing siliques (S1-S4). L22 and L24 represent two independent E2F-binding-site-mutant promoter lines (D). Values represent fold changes normalised to the value of the intact promoter construct at the S1 silique stage (set arbitrarily at 1). Data are mean±s.d., *n*=3 biological repeats. *P≤0.05, **P≤0.01 (two-tailed, paired *t*-test between the corresponding mutant and the intact promoter construct, at a given silique stage). ns, non-significant. (E) Immunoblot assay using anti-GFP antibody showing the CFP protein level in developing siliques (S1-S3) of the same transgenic lines shown in D, as indicated. Molecular weight of the CFP protein (28 kDa) is indicated on the left. The Coomassie-stained proteins were used as loading control.

The promoter regions of *LEC2* and *WRI1* have putative E2F-binding sites, suggesting that E2Fs may directly control their expression (Fig. 6A). To test this, we performed chromatin immunoprecipitation (ChIP) experiments with anti-GFP antibody on silique samples collected from the maturation phase (S3) of pgE2FA-GFP and pgE2FB-GFP lines (Magyar et al., 2012). We could detect significant enrichment of E2FA-GFP but not E2FB-GFP protein to the promoter of *LEC2*, and neither protein is detected at the *WRI1* promoter (Fig. 6B; Fig. S7). E2FA-GFP enrichment on the *LEC2* promoter was located specifically to the region in which the consensus E2F-binding element was predicted to be (Fig. 6A,B). This result suggested that E2FA could directly regulate the expression of *LEC2* during the maturation phase. This experiment cannot rule out whether E2FC has a role during the S3 stage or whether there are E2F associations during earlier seed developmental phases, which is not amenable for ChIP. To gain further evidence for the E2F-mediated regulation of genes during seed maturation, we mutated the putative E2F-binding site identified in the promoter regions of *LEC2* and *WRI1*. We generated reporter lines expressing the cyan fluorescent protein (CFP) either under the control of the native or the E2F-binding-site mutant *LEC2* and *WRI1* promoters. Representative lines were selected and siliques were harvested at different developmental stages, as before (S1-S4). The intact LEC2 promoter-reporter line (pLEC2-CFP) showed similar expression pattern as the endogenous *LEC2* transcript (Fig. 1A); there was almost no *LEC2* expression in the earliest seed developmental phase (S1), but it increased in the transition S2 phase, reached the maximum level in the maturation phase (S3) and diminished afterwards in post-mature seeds (S4; Fig. 6C). In contrast, the E2F-site mutant LEC2 promoter-reporter line (p^mutE2F^LEC2-CFP) showed an elevated and nearly constitutive transcript level throughout the silique development stages (Fig. 6C). The WRI1 promoter-reporter line (pWRI1-CFP) also closely followed the endogenous *WRI1* expression, peaking during the maturation (S3) phase (Figs 1A and 6D). We analysed two independent E2F-binding site mutant WRI1 promoter-reporter lines (p^mutE2F^WRI1-CFP, lines 22 and 24). Both of these reporter lines were expressed prematurely in the early developmental phases of S1-S2, line 24 to a larger extent than line 22 (Fig. 6D). To back up these results we also monitored the CFP protein levels in these pWRI1 reporter-CFP lines during silique development (Fig. 6E). In the intact pWRI1-CFP line, CFP was exclusively accumulated at high level during the maturation phase (S3), whereas CFP protein could be detected in the earlier developmental silique stages in both p^mutE2F^WRI1-CFP lines (Fig. 6E). These data further support that the timing of expression for these maturation genes is regulated by E2Fs.

**Fig. 7.**
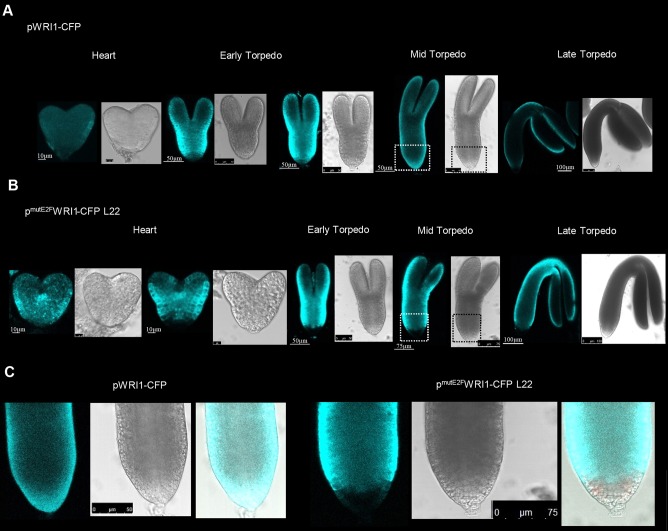
**The temporal and spatial expression of** ***WRI1*** **in the developing embryo depend on its regulatory E2F-binding site.** (A,B) Representative confocal images of developing embryos from the intact (A) and E2F-binding site mutant (B) *WRI1* promoter (pWRI1::CFP and p^mutE2F^WRI1::CFP reporter lines, respectively) dissected from immature seeds. (C) Magnifications of dashed boxed areas in A (left) and B (right) showing the hypocotyl-root tip regions of mid-torpedo stage embryos. CFP signal (blue, left), bright field (middle) and the merged (right) images are shown.

Contrary to the reporter pLEC2-CFP lines, the pWRI1-CFP signal was high enough to allow confocal microscopy detection in the developing embryos. Confirming previous findings, in the pWRI1-CFP line the fluorescence signal was hardly detectable in the heart-stage embryo, being the brightest at the beginning of maturation phase in the early torpedo embryo stage, gradually declining afterwards during maturation and diminishing in the fully mature embryo (Fig. 7A; Baud et al., 2007). In contrast, both p^mutE2F^WRI1-CFP line 22 (Fig. 7B) and line 24 (Fig. S8) showed a strong CFP signal in the heart-stage embryo, which was maintained at a high level until the mid- and late-torpedo embryo stages (Fig. S8). Although the CFP signal was stronger for a longer period of time in the p^mutE2F^WRI1-CFP lines, the signal was missing in the root tip region of the immature embryos in comparison with the pWRI1-CFP line (Fig. 7C; Fig. S8B), suggesting that E2Fs both temporally and spatially regulate the expression of WRI1 during embryogenesis.

### Seed reserve accumulation is prematurely activated in the *e2fab* double mutant

**Fig. 8.**
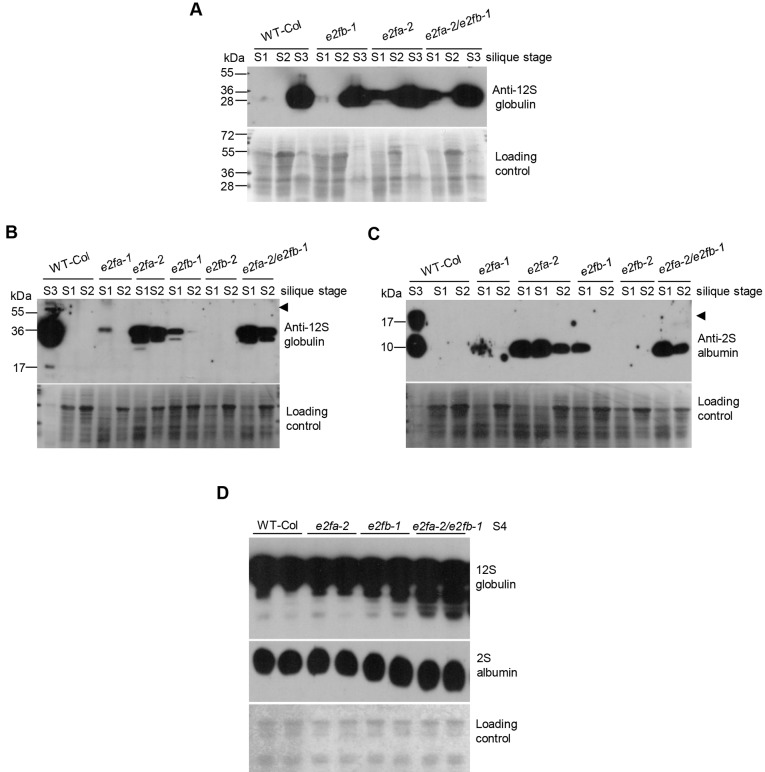
**Seed storage proteins 2S albumin and 12S globulin show premature accumulation in the *e2f* mutant siliques and seeds.** (A) Accumulation levels of 12S globulin in the single *e2fa-2*, *e2fb-1* and in the double *e2fa-2/e2fb-1* mutants at three silique and seed developmental stages (S1-S3) were compared with the control WT in immunoblot assay using a specific antibody. (B,C) SSPs were detected in the early developing siliques and seeds of *e2fa-1*, *e2fa-2*, *e2fb-1* and *e2fb-2* single, as well as *e2fa-2/e2fb-1* double mutants by using anti-12S (B) or anti-2S (C) antibodies in immunoblot assays. The WT maturation-phase silique sample (S3) was used as positive control, and a quarter of the S1-S2 samples were analysed (5 µg). Arrowheads indicate precursors of 12S or 2S proteins. (D) The amount of globulin (12S) and albumin (2S) in post-mature seeds (S4) of WT, single and double activator *e2f* mutants were compared using specific anti-12S and anti-2S antibodies in immunoblot assays. Coomassie-stained proteins were used as loading controls.

The results presented so far indicated that E2FA and E2FB repress key maturation genes during seed and silique development, which prompted us to test whether these activator E2Fs could regulate the seed maturation programme. The two major seed storage proteins (SSPs) are the globulin (12S) and the albumin (2S) that represent up to one third of the dry weight in *Arabidopsis* seeds (Baud et al., 2002). To study the role of activator E2Fs, we determined the 2S albumin and 12S globulin levels during silique and seed development in single and double *e2fa* and *e2fb* mutants (Fig. 8). As known, these SSPs exclusively accumulate during the maturation phase (S3) of the control WT siliques (Fig. 8A; Vicente-Carbajosa and Carbonero, 2005), but became considerably more abundant in the S1 stage in *e2fa-2* and to a lower degree in the *e2fb-1* mutants, whereas the upregulation in double *e2fab* mutants was comparable with that of *e2fa-2* at the S1 stage (Fig. 8A-C). The position of the transfer DNA (T-DNA) insertion in the *e2fa-2* allele is just after the MARKED-Box (MB), whereas for *e2fb-1* it is after the dimerisation domain (DD, Fig. S9A). The MB domain strengthens the interaction with DPs directed by the dimerisation domain, which is a requirement to bind the target promoters (Black et al., 2005; Rubin et al., 2005). To address whether the upregulation of SSPs is differently affected by *e2fa* or *e2fb* mutations, or correlates with the site of T-DNA insertion and possible production of truncated proteins with different properties, we analysed our mutant collection of *e2fa* and *e2fb* alleles (Fig. S9A). First we confirmed by qRT-PCR using insertion-surrounding primers that T-DNA insertion is present in these mutants (Fig. S9B,E), and with 5′-specific primers we could detect both *E2FA* and *E2FB* transcripts in the mutants (Fig. S9F,G). Using RT-PCR with primer pairs spanning the T-DNA, we further confirmed that these *e2f* mutants produce transcripts down to the insertion sites (Fig. S10A,B), although using primers downstream of the insertion could not amplify any fragments (Fig. S10C,D). Using an E2FB antibody targeted to the C-terminus, we established that there is neither full-length nor truncated E2FB proteins containing part of the C-terminus in the *e2fb-1* and *e2fb-2* mutants (Fig. S11A). To test for the existence of a truncated E2FB protein, we used an N-terminal-specific E2FB antibody. This antibody is specific for recognising the overexpressed E2FB-GFP, but too weak to detect the endogenous E2FB, unless it was enriched through DPA co-immunoprecipitation (Fig. S11C), and in this way we could confirm the existence of a truncated E2FB protein in the *e2fab* double mutant (Fig. S11D). By using an N-terminal-specific E2FA antibody, the full-length protein was recognised in WT, *e2fb-1* and *e2fb-2* mutants, and a smaller sized protein was detected in the *e2fa-2* single and *e2fab* double mutants (Fig. S11B). This protein could not be observed in *e2fa-1*, supporting that this is a truncated E2FA specific for the *e2fa-2* mutation. These results support that the *e2fa* and *e2fb* T-DNA insertion mutants can produce truncated proteins that expect to affect RBR recruitment and transactivation. In addition, these truncated proteins may have a different ability to bind to DNA: the MB domain is intact in *e2fa-2*, which should allow strong DNA binding; the T-DNA insertion disrupts the MB region in *e2fa-1* and *e2fb-1* at a comparable position, which is expected to weaken their DNA binding activities; and the dimerisation domain is disrupted in the *e2fb-2*, which should prevent DNA binding. With this in mind, we went on to determine how these different *e2fa* and *e2fb* mutant alleles affect the accumulation of 12S globulin and 2S albumin protein at different stages of silique development. These storage proteins are only present at the mature S3 stage in WT. In contrast, they were prematurely accumulated in these *e2f* mutants except in *e2fb-2* (Fig. 8B,C). Interestingly, the extent of premature expression of these storage proteins followed the predicted binding of truncated E2FA or E2FB to DNA as it was the strongest in *e2fa-2* as well as in the double *e2fab*, weaker in *e2fb-1* and *e2fa-1*, and no effect was seen in *e2fb-2*. This suggests that the binding of these E2F mutant proteins to target DNA sequences without the ability to recruit the repressor RBR protein is what leads to the premature expression of SSPs.

Because SSPs started to accumulate earlier during seed development in the *e2f* mutants, we wondered whether they reached higher levels in the fully developed post-mature dry seeds than in WT. We found that in the single *e2fa-1* and *e2fb-1* mutants both 2S albumin and 12S globulin accumulated to comparable levels as those seen in the WT, whereas the 12S globulin became more abundant in the *e2fab* double mutant seeds (Fig. 8D). We also determined the total protein content in mature seeds and, as shown in Table 2, it was significantly higher than the WT in the *e2fab* mutant. Thus, the embryo of the *e2fab* double mutant might become larger than WT because it contains more seed storage reserves.

**Table 2.**
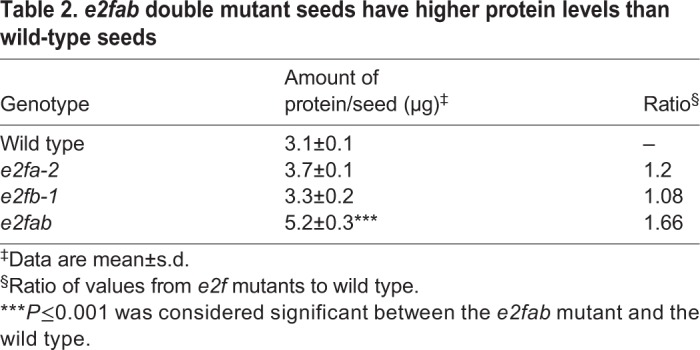
***e2fab*** **double mutant seeds have higher protein levels than wild-type seeds**

**Fig. 9.**
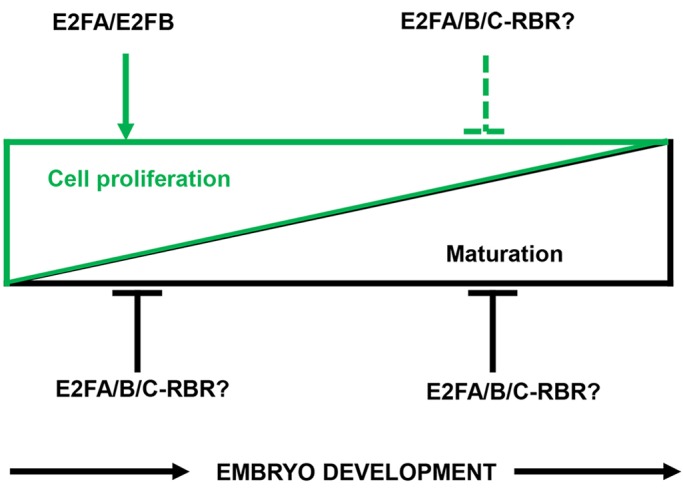
**Model explaining the functions of activator E2Fs during seed and embryo development.** The proliferative morphogenic (green triangle) and the differentiation-related maturation (black triangle) phases are the two major and oppositely regulated phases of seed and embryo development. Activator E2Fs are required for the full activation of cell cycle genes in the morphogenic developmental phase, whereas in the subsequent maturation phase they are involved in the repression of cell proliferation, probably together with E2FC and in complex with RBR to establish quiescence. The maturation programme is inhibited in the proliferative phase by activator E2Fs through either repression of the expression of maturation genes such as *LEC2* or inhibition of the accumulation of the SSPs 2S albumin and 12S globulin. Activator E2Fs also tune the expression of maturation genes during the differentiation phase of seed development, and E2FC and RBR might also participate in this regulation.

In summary, we uncovered an important regulatory function for the activator E2Fs during the early morphogenic seed developmental phase to restrict the maturation programme until proliferation is active (Fig. 9).

## DISCUSSION

Here, we showed that the two activator E2Fs, E2FA and E2FB, coordinate cell proliferation with differentiation during seed and embryo development through multiple mechanisms: (1) both are contributing to the expression of cell cycle genes in the early phases of embryo development, but they are not essential for cell proliferation; (2) they have distinct roles to repress S- and M-phase genes during seed maturation, when embryo quiescence is established; (3) these activator E2Fs also have distinct roles to repress embryonic-differentiation genes including *LEC2* and *WRI1*; (4) these E2F transcription factors are crucial for the timing and extent of SSP accumulation (Fig. 9).

### E2FA and E2FB mutations do not affect cell number in the developing embryo

The expression of S-phase-specific genes was not affected in the single *e2fa-2* and *e2fb-1* mutants, but it was in the double *e2fab* mutant, indicating that the mutations act redundantly on S-phase regulatory genes. In contrast, the mitotic *CDKB1;1* was exclusively regulated by E2FB but not by E2FA. In agreement, *E2FB* but not *E2FA* is expressed during the G2/M phases of the cell cycle (Mariconti et al., 2002; Magyar et al., 2005). The moderate overexpression of E2FA upregulates S-phase specific genes, whereas the ectopic expression of CYCD3;1 hyper-activates both S- and M-phase regulatory genes, similar to E2FB (de Jager et al., 2009). Moreover, it was suggested that E2FB is the canonical cell cycle activator E2F in *Arabidopsis*, based on the finding that it is released from RBR repression in the CYCD3;1 overexpressor line, whereas the E2FA-RBR complex was found to be regulated differently (Magyar et al., 2012).

In spite of the partial requirement for these activator E2Fs to fully promote cell cycle genes, the double *e2fab* mutant embryos consist of a number of cells comparable with the control WT. These findings demonstrate that E2FA and E2FB are partially required but not essential for the expression of cell cycle target genes during embryonic cell divisions and the reduced expression of these cell cycle genes does not manifest in reduced cell proliferation. This is in agreement with other results showing that the regulatory roles for activator E2Fs are not essential for meristematic cell proliferation during post-embryonic development (Wang et al., 2014). Together with findings in animal systems, a universal model is emerging in which activator E2F functions are not required for normal cell proliferation either in embryonic or in post-embryonic development, which holds both for animals and for plants (Rowland and Bernards, 2006; Chen et al., 2009a,b; Chong et al., 2009; Magyar et al., 2016; Zappia and Frolov, 2016).

We could not detect developmental abnormalities in the *e2f* mutant embryos, except the significantly enlarged seed and embryo size in the double *e2fab* mutant. Interestingly, the double *e2fab* mutant develops shorter siliques containing fewer seeds than the control, and we found that *e2fab* has compromised fertility. It was shown that fertility problems might account for 33% of the increase in average seed weight (Ohto et al., 2005). This value matches the increase we observed with the *e2fab* double mutant. In agreement with the lack of cell proliferation defects, the plant stature of the *e2fab* double mutant does not differ from the WT during post-embryonic development. Recently, it was suggested that the three *Arabidopsis* E2Fs regulate germline development in a redundant manner and affect fertility both through pollen development and megaspore mother cells (Yao et al., 2018). Indeed, the triple *e2fabc* mutant plants hardly produce seeds, but the plant stature is seemingly unaffected (Wang et al., 2014; Yao et al., 2018). Thus, none of the three canonical plant E2Fs is essential for cell proliferation, at least during the sporophyte development. There are indications that some of the non-canonical E2Fs, i.e. E2FD, may have positive roles in cell proliferation (Sozzani et al., 2010), possibly by competing with repressor complexes at E2F sites when the canonical E2Fs are missing.

### E2FA and E2FB function as repressors in post-mitotic embryonic cells during maturation

Cell cycle genes are turned off during the transition phase from proliferation to maturation in the developing embryo, but the molecular mechanism underlying this is not yet clear. Here, we show that cell cycle genes remained partially on even after the completion of the proliferation phase in the double *e2fab* mutant. This shows that these two E2Fs function as repressors on cell cycle genes as seed development progresses into the maturation phase. It is likely that E2Fs form a complex with the transcriptional repressor RBR protein at this phase of seed development to establish quiescence, as has previously been shown during seedling and leaf development (Kobayashi et al., 2015). In agreement, E2FA and E2FB, as well as their upstream regulator RBR proteins, are present in post-mitotic embryonic cells. The cell number in *rbr* mutant embryos increases during the maturation phase (Gutzat et al., 2011). However, in the *e2fab* mutant embryo we did not see a significant increase in cell number, indicating the requirement for additional components besides E2FA and E2FB downstream of RBR, likely E2FC, to repress cell proliferation during the maturation phase of embryogenesis.

These data support that RBR is essential for determining the cell number in developing embryos and other plant organs in close association with E2Fs through the formation of repressor complexes. Interestingly, when RBR level or activity are reduced in plants the result is hyper-proliferation and tumorous growth, as is seen when *Rb* is eliminated in animals (Borghi et al., 2010; Gutzat et al., 2011; Chen et al., 2009b). The simultaneous inactivation of activator E2F1-3 in *rb* mutant animals restores normal development, indicating that animal E2F activator function is essential for tumour development, but dispensable for normal proliferation (Chen et al., 2009b). In plants it remains to be demonstrated whether the elimination of E2Fs in lines in which *rbr* is compromised could restore the normal proliferation rate. Because RBR was also shown to be the primary target of CDKA;1 (Nowack et al., 2012), it would be also interesting to examine whether the elimination of E2Fs in *cdka;1* mutants could restore the embryo defect.

### Activator type E2Fs function as repressors to regulate the timing of the embryo maturation programme in developing seeds

Loss-of-function mutations in the LEC genes cause a defect in reserve accumulation (Braybrook and Harada, 2008). We found that both LEC genes were prematurely upregulated in the *e2fab* double mutant. In addition, we show that the *LEC2* gene could be directly regulated by E2Fs through an E2F-binding site during the maturation phase. In addition, *LEC2* expression was also prematurely activated in the *e2fb-1* mutant, suggesting that E2FB regulates *LEC2*, but earlier than E2FA. In agreement, expression of *LEC2* became de-regulated when the E2F site in the promoter was mutated, and showed a nearly maximum level of expression during the morphogenic developmental phase. We also studied another putative E2F target gene, *WRI1*, and showed that it is prematurely activated when the E2F-binding site was mutated. These data point to E2Fs as negative regulators of maturation genes, not just limiting their expression while cell proliferation is ongoing, but also fine tuning their expressions during the maturation phase. In young *Arabidopsis* seedlings of an *rbr* co-suppression line the maturation genes, including *LEC2* and *ABI3*, remain active, indicating that RBR controls these genes during post-embryonic development (Gutzat et al., 2011). It is also known that these maturation genes are under the control of the Polycomb group (PcG) (Yang et al., 2013). Whether E2Fs together with RBR are also involved in this repressor complex remains to be seen.

We found that the major SSPs 12S globulin and 2S albumin have already, prematurely accumulated at the morphogenic developmental phase in seeds of *e2fa-2*, *e2fb-1* and *e2fab* mutants. Interestingly, at this early time point none of the regulatory AFL genes of seed maturation was upregulated in these *e2fa* and *e2fb* mutants, suggesting that these are not involved in the observed advance in the accumulation of SSPs. E2FA has previously been found to repress the switch from mitosis to endocycle during leaf development by forming a repressor complex with RBR (Magyar et al., 2012). Simultaneous overexpression of E2FA with its dimerisation partner, DPA, delays differentiation during early seedling development (De Veylder et al., 2002; Kosugi and Ohashi, 2003). The data presented here show that E2FA is a potential repressor of the developmental transition programme of seed maturation, suggesting that this developmental role of E2FA is more general. Whether E2FA performs this repressive role in a complex with RBR is not yet known. Both E2FA and RBR proteins clearly accumulate at the highest level in the morphogenic seed developmental phase, supporting the hypothesis that they can form a complex at this early seed developmental stage. Interestingly, the accumulation of SSPs was less pronounced in the *e2fa-1* and *e2fb-1* mutants in comparison with the *e2fa-2*, and was not observed in the *e2fb-2*. We confirmed that truncated proteins can be produced until the T-DNA insertion. As all these truncated proteins are predicted to lack the ability to bind RBR or to transactivate, the difference between these alleles could be their ability to bind DNA; however, this needs to be experimentally verified. It is possible that the truncated E2FA mutant product occupies the binding sites and thus prevents the formation of other repressor complexes. Accordingly, all three E2Fs, including E2FC, and possibly also the non-canonical E2Fs (the DELs) might regulate the timing of seed maturation.

In conclusion, the RBR-E2F network is important both for the extent of seed growth and accumulation of seed storage reserves and should be considered as an important breeding target to increase crop yield.

## MATERIALS AND METHODS

### Plant material, growth conditions and silique collection

*Arabidopsis thaliana* Col-0 ecotype was the WT and background of every transgenic line used in this study. *In vitro*-cultured plants were grown on half-strength germination medium under continuous light at 22°C. Soil-grown plants were cultivated in a greenhouse at 22°C under long-day conditions (16 h light/8 h dark). All the T-DNA insertion mutant lines used in the experiments have been previously published: *e2fb-1*, SALK_103138; *e2fb-2*, SALK_120959; *e2fa-1*, MPIZ-244, *e2fa-2*, GABI-348E09 (Berckmans et al., 2011a,b; Horvath et al., 2017); the double *e2fab* was reported by Heyman et al. (2011). Total seed weight, seed size, number of siliques on the main inflorescence, seed number per siliques and silique size were measured using ten plants per genotype according to Van Daele et al. (2012). Seed size was calculated from 100 seeds imaged by stereo microscope and analysed by ImageJ software (Schneider et al., 2012).

Siliques were collected from soil-grown plants at four different developmental stages: S1, young siliques 2-3 days after pollination (DAP), 0.2-0.3 cm length; S2, siliques 4-7 DAP, 0.4-0.6 cm size; S3, full-size siliques 8-12 DAP; S4, full-size yellow siliques, 13-18 DAP.

### Generation of reporter lines and transgenic *Arabidopsis* plants

Transgenic lines expressing the 3xvYFP-tagged E2FA (pgE2FA-3xvYFP) or E2FB (pgE2FB-3xvYFP) have been recently described (O˝szi et al., 2019). The genomic sequence of RBR was fused in frame with 3xCFP in a pGreenII-based pGII0125 destination vector by using the Invitrogen 3way Gateway System (Thermo Fisher Scientific). To construct the transcriptional reporters pLEC2-CFP and pWRI1-CFP, promoter regions of *LEC2* and *WRI1* genes were PCR amplified (3162 bp and 1864 bp upstream of the translational start codon, respectively; cloning primer combinations described in Table S1). The Multisite Gateway cloning strategy was used to make promoter-reporter gene fusions following the protocols in the Gateway Cloning Technology booklet (Thermo Fisher Scientific). The *LEC2* and *WRI1* promoter regions were cloned into pGEM-based plasmids and, together with the CFP reporter in the pGEM 221 plasmid, introduced to a pGreenII-based pGII0229 destination vector. Site-directed mutagenesis was carried out using the QuikChange mutagenesis system (Stratagene; Papworth et al., 1996). The E2F-binding site TTTCCCCC on the *WRI1* promoter at the −359 bp position was mutated to TTTCCAAC and the CGGGAAAA motif on the *LEC2* promoter at the −2 bp position was mutated to TTGGAAAA. Primers used for the mutagenesis are described in Table S1. Transgenic *Arabidopsis* plants were generated using the floral-dip method for *Agrobacterium*-mediated transformation and primary transformants were selected on soil by spraying BASTA (300 mg/l glufosinate-ammonium; Finale 14SL, Bayer Crop Science). We identified 24 pWRI1-CFP, 31 p^mutE2F^WRI1-CFP, 26 pLEC2-CFP and 24 p^mutE2F^LEC2-CFP primary transgenic lines, which were genotyped by phosphinothricin selection (PPT, Dutchefa). Single insertion lines were identified and used for further analysis (five in each case).

### RNA extraction and qRT-PCR

Total RNA was extracted from siliques and developing seeds using the CTAB-LiCl method (Jaakola et al., 2001). Isolated RNA samples were treated with DNaseI (Thermo Fisher Scientific), and 1 μg of RNA was used to prepare cDNA from each sample using the RevertAid First Strand cDNA Synthesis Kit (Thermo Fisher Scientific). qRT-PCR was performed using Maxima SYBR Green/ROX qPCR Master Mix (2×) (Thermo Fisher Scientific) and the Applied Biosystems 7900-HT Fast Real-Time detection system. For amplification, a standard two-step thermal cycling profile was used (15 s at 95°C and 1 min at 60°C) for 40 cycles, after a 10-min preheating step at 95°C. Samples were run in triplicates, and *UBC18* was used as the internal reference gene. Data analysis was carried out using either the 2-ΔCT or the 2-ΔΔCT method. The Student's *t*-test was used to determine the significance of differences between groups. Data are presented as mean±s.d.

### Protein analysis, protein extraction, antibody preparation and immunoblot assay

Siliques were collected from different developmental stages (40-50 siliques per line) and snap-frozen in liquid nitrogen and stored at −40°C. For detecting E2F-DP and cell cycle proteins in immunoblot assay, total proteins were extracted from developing immature siliques (stage S1-S3) in extraction buffer [25 mM TRIS-HCl, 15 mM MgCl_2_, 15 mM EGTA, 15 mM p-nitrophenylphosphate, 100 mM NaCl, 60 mM ß-glycerophosphate, 1 mM DTT, 0.1% Igepal, 5 mM NaF, protease inhibitor cocktail for plant tissue (Sigma-Aldrich) and 1 mM phenylmethyl sulphonylfluoride (Magyar et al., 1993)], and total proteins from post-maturing siliques (S4) or 100 dry seeds were extracted in extraction buffer in a mortar cooled in liquid nitrogen [100 mM Tris-HC1 (pH 8.0), 0.5% (w/v) SDS, 10% (v/v) glycerol and 2% (v/v) 2-mercaptoethanol (Hou et al., 2005)], this time followed by boiling for 3 min, and centrifugation for 10 min at 4°C (17,000 ***g***). The latter extraction method was used for detecting SSPs in siliques of S1 to S4 stages. The precipitated material (20-40 µg) was separated on SDS-PAGE (10%, 12% or 15%) and either stained by Coomassie-Brilliant Blue R250 or blotted to a PVDF membrane. Antibodies used in the immunoblotting experiments were: chicken polyclonal anti-RBR (1:2000; Agrisera, AS111627), rat polyclonal antibody anti-E2FA (1:300; see below), rabbit polyclonal antibody anti-E2FB, (1:500; Magyar et al., 2005), N-terminal specific chicken polyclonal anti-E2FB (1:300; see below), rabbit polyclonal anti-DPB (1:500; López-Juez et al., 2008), rabbit polyclonal antibody anti-2S albumin and anti-12S globulin (both 1:10,000; Shimada et al., 2003).

To produce the E2FA antibody, a 270 bp fragment encoding the N-terminal 90 amino acids of *Arabidopsis* E2FA (E2FA-N-90) was amplified using the following primers: BamHI-FWD: 5′-ATAGGATCCATGTCCGGTGTCGTACGATC-3′; SalI-REV: 5′-ATAGTCGACCTATCTAACAACGACAGCATCTTCCT-3′ (restriction sites underlined). The BamHI-SalI-digested E2FA-N-90 fragment was subcloned into the pET-28a(+) vector (Novagen) to obtain 6×His-E2FA-N-90 and this construct was transformed into BL21(DE3) Rosetta cells (Novagen). Protein production was induced with 0.5 mM IPTG (3 h, 37 C, 250 rpm shaking), cells were lysed in 6 M GuHCl lysis buffer and the cleared lysate was loaded onto HIS-Select Nickel Affinity Gel (Sigma-Aldrich, P6611). The 6×His-E2FA-N-90 protein was purified according to the manufacturer's (Novagen) instructions and used to immunise rats. The immunoglobulin fraction of crude rat sera was obtained by ammonium-sulfate precipitation, anti-E2FA antibody was further purified on nitrocellulose-bound recombinant protein following the protocol in Kurien (2009).

To produce the N-terminal-specific E2FB antibody, a 267 bp fragment encoding the N-terminal 89 amino acids was amplified using the following primers: BamHI-FWD: 5′- ACGGATCCATGTCTGAAGAAGTACCT-3′; Sal1-REV: 5′ ATAGTCGACTGATACAGGTGTTTGAAG-3′ (restriction sites underlined). The PCR E2FB-N-89 fragment was cloned into the pGEX-4T-1 vector (GE Healthcare Life Sciences) into the BamH1-Sal1 sites, and the recombinant GST-tagged E2FB-N-89 protein was purified after IPTG induction according to the manufacturer's instructions (GE Healthcare Life Sciences) and used to immunise chickens. The antibody was further purified as for the anti-E2FA antibody (see above).

### Chromatin immunoprecipitation

The ChIP assay was carried out according to Saleh et al. (2008). We crosslinked 2 g of siliques from developmental stage S3 of E2FA-GFP (Berckmans et al., 2011b) or E2FB-GFP expressing plants with 1% formaldehyde solution at 6 days after germination (DAG). Chromatin was precipitated using anti-GFP polyclonal rabbit antibody (1:125; Invitrogen, A-11122) and collected with salmon sperm DNA/protein A-agarose (Sigma-Aldrich). The purified DNA was used in qRT-PCR reactions to amplify promoter regions with the specific primers listed in Table S1. Relative DNA enrichment was calculated by dividing the antibody immunoprecipitation signals with the no-antibody signals.

### Dissecting of embryos, and microscopy

Immature embryos of transgenic lines expressing the fluorescent tagged E2FA, E2FB or RBR proteins under the control of their own promoters (pgE2FA-3xvYFP, pgE2FB-3xvYFP or pgRBR-3xCFP) were dissected under a stereo-microscope (Olympus, SZX12), and observations were made using a Leica confocal laser microscope (Leica SP5). Mature dried seeds were imbibed for 1 h and dissected under the stereo-microscope. Isolated embryos were stained with PI and photographed. Organ and epidermal cell sizes were measured using ImageJ software (Schneider et al., 2012).

## Supplementary Material

Supplementary information

## References

[DEV179333C1] Baud S., Boutin J.-P., Miquel M., Lepiniec L. and Rochat C. (2002). An integrated overview of seed development in *Arabidopsis thaliana* ecotype WS. *Plant Physiol. Bioch.* 40, 151-160. 10.1016/S0981-9428(01)01350-X

[DEV179333C2] Baud S., Mendoza M. S., To A., Harscoët E., Lepiniec L. and Dubreucq B. (2007). WRINKLED1 specifies the regulatory action of LEAFY COTYLEDON2 towards fatty acid metabolism during seed maturation in *Arabidopsis*. *Plant J.* 50, 825-838. 10.1111/j.1365-313X.2007.03092.x 17419836

[DEV179333C3] Baud S., Dubreucq B., Miquel M., Rochat C. and Lepiniec L. (2008). Storage reserve accumulation in *Arabidopsis*: metabolic and developmental control of seed filling. *Arabidopsis Book* 6, e0113 10.1199/tab.0113 22303238 PMC3243342

[DEV179333C4] Berckmans B., Lammens T., Van Den Daele H., Magyar Z., Bögre L. and De Veylder L. (2011a). Light-dependent regulation of DEL1 is determined by the antagonistic action of E2Fb and E2Fc. *Plant Physiol.* 157, 1440-1451. 10.1104/pp.111.183384 21908689 PMC3252145

[DEV179333C5] Berckmans B., Vassileva V., Schmid S. P. C., Maes S., Parizot B., Naramoto S., Magyar Z., Alvim Kamei C. L., Koncz C., Bögre L. et al. (2011b). Auxin-dependent cell cycle reactivation through transcriptional regulation of *Arabidopsis* E2Fa by lateral organ boundary proteins. *Plant Cell* 23, 3671-3683. 10.1105/tpc.111.088377 22003076 PMC3229142

[DEV179333C6] Black E. P., Hallstrom T., Dressman H. K., West M. and Nevins J. R. (2005). Distinctions in the specificity of E2F function revealed by gene expression signatures. *Proc. Natl. Acad. Sci. USA* 102, 15948-15953. 10.1073/pnas.0504300102 16249342 PMC1276052

[DEV179333C7] Boniotti M. B. and Gutierrez C. (2001). A cell-cycle-regulated kinase activity phosphorylates plant retinoblastoma protein and contains, in *Arabidopsis*, a CDKA/cyclin D complex. *Plant J.* 28, 341-350. 10.1046/j.1365-313x.2001.01160.x 11722776

[DEV179333C8] Borghi L., Gutzat R., Fütterer J., Laizet Y., Hennig L. and Gruissem W. (2010). *Arabidopsis* RETINOBLASTOMA-RELATED is required for stem cell maintenance, cell differentiation, and lateral organ production. *Plant Cell* 22, 1792-1811. 10.1105/tpc.110.074591 20525851 PMC2910961

[DEV179333C9] Braybrook S. A. and Harada J. J. (2008). LECs go crazy in embryo development. *Trends Plant Sci.* 13, 624-630. 10.1016/j.tplants.2008.09.008 19010711

[DEV179333C10] Carbonero P., Iglesias-Fernández R. and Vicente-Carbajosa J. (2017). The AFL subfamily of B3 transcription factors: evolution and function in angiosperm seeds. *J. Exp. Bot.* 68, 871-880. 10.1093/jxb/erw458 28007955

[DEV179333C11] Chen D., Pacal M., Wenzel P., Knoepfler P. S., Leone G. and Bremner R. (2009a). Division and apoptosis of E2f-deficient retinal progenitors. *Nature* 462, 925-929. 10.1038/nature08544 20016601 PMC2813224

[DEV179333C12] Chen H.-Z., Tsai S.-Y. and Leone G. (2009b). Emerging roles of E2Fs in cancer: an exit from cell cycle control. *Nat. Rev. Cancer* 9, 785-797. 10.1038/nrc2696 19851314 PMC3616489

[DEV179333C13] Chong J.-L., Wenzel P. L., Sáenz-Robles M. T., Nair V., Ferrey A., Hagan J. P., Gomez Y. M., Sharma N., Chen H.-Z., Ouseph M. et al. (2009). E2f1-3 switch from activators in progenitor cells to repressors in differentiating cells. *Nature* 462, 930-934. 10.1038/nature08677 20016602 PMC2806193

[DEV179333C14] Collins C., Dewitte W. and Murray J. A. H. (2012). D-type cyclins control cell division and developmental rate during *Arabidopsis* seed development. *J. Exp. Bot.* 63, 3571-3586. 10.1093/jxb/ers015 22412186 PMC3388828

[DEV179333C15] de Jager S. M., Scofield S., Huntley R. P., Robinson A. S., den Boer B. G. W. and Murray J. A. H. (2009). Dissecting regulatory pathways of G1/S control in *Arabidopsis*: common and distinct targets of CYCD3;1, E2Fa and E2Fc. *Plant Mol. Biol.* 71, 345-365. 10.1007/s11103-009-9527-5 19662336

[DEV179333C16] del Pozo J. C., Boniotti M. B. and Gutierrez C. (2002). *Arabidopsis* E2Fc functions in cell division and is degraded by the ubiquitin-SCF(AtSKP2) pathway in response to light. *Plant Cell* 14, 3057-3071. 10.1105/tpc.006791 12468727 PMC151202

[DEV179333C17] del Pozo J. C., Diaz-Trivino S., Cisneros N. and Gutierrez C. (2006). The balance between cell division and endoreplication depends on E2FC-DPB, transcription factors regulated by the ubiquitin-SCFSKP2A pathway in *Arabidopsis*. *Plant Cell* 18, 2224-2235. 10.1105/tpc.105.039651 16920782 PMC1560920

[DEV179333C18] De Veylder L., Beeckman T., Beemster G. T. S., de Almeida Engler J., Ormenese S., Maes S., Naudts M., Van Der Schueren E., Jacqmard A., Engler G. et al. (2002). Control of proliferation, endoreduplication and differentiation by the *Arabidopsis* E2Fa-DPa transcription factor. *EMBO J.* 21, 1360-1368. 10.1093/emboj/21.6.1360 11889041 PMC125359

[DEV179333C19] De Veylder L., Beeckman T. and Inzé D. (2007). The ins and outs of the plant cell cycle. *Nat. Rev. Mol. Cell Biol.* 8, 655-665. 10.1038/nrm2227 17643126

[DEV179333C20] Devic M. and Roscoe T. (2016). Seed maturation: simplification of control networks in plants. *Plant Sci.* 252, 335-346. 10.1016/j.plantsci.2016.08.012 27717470

[DEV179333C21] Fischer M. and DeCaprio J. A. (2015). Does *Arabidopsis thaliana* DREAM of cell cycle control? *EMBO J.* 34, 1987-1989. 10.15252/embj.201592196 26089020 PMC4551346

[DEV179333C22] Focks N. and Benning C. (1998). wrinkled1: A novel, low-seed-oil mutant of *Arabidopsis* with a deficiency in the seed-specific regulation of carbohydrate metabolism. *Plant Physiol.* 118, 91-101. 10.1104/pp.118.1.91 9733529 PMC34877

[DEV179333C23] Goldberg R. B., de Paiva G. and Yadegari R. (1994). Plant embryogenesis: zygote to seed. *Science* 266, 605-614. 10.1126/science.266.5185.605 17793455

[DEV179333C24] Gutierrez C. (2009). The *Arabidopsis* cell division cycle. *Arabidopsis Book* 7, e0120 10.1199/tab.0120 22303246 PMC3243301

[DEV179333C25] Gutzat R., Borghi L., Futterer J., Bischof S., Laizet Y., Hennig L., Feil R., Lunn J. and Gruissem W. (2011). RETINOBLASTOMA-RELATED PROTEIN controls the transition to autotrophic plant development. *Development* 138, 2977-2986. 10.1242/dev.060830 21693514

[DEV179333C26] Gutzat R., Borghi L. and Gruissem W. (2012). Emerging roles of RETINOBLASTOMA-RELATED proteins in evolution and plant development. *Trends Plant Sci.* 17, 139-148. 10.1016/j.tplants.2011.12.001 22240181

[DEV179333C27] Harashima H. and Sugimoto K. (2016). Integration of developmental and environmental signals into cell proliferation and differentiation through RETINOBLASTOMA-RELATED 1. *Curr. Opin. Plant Biol.* 29, 95-103. 10.1016/j.pbi.2015.12.003 26799131

[DEV179333C28] Heyman J., Van den Daele H., De Wit K., Boudolf V., Berckmans B., Verkest A., Alvim Kamei C. L., De Jaeger G., Koncz C. and De Veylder L. (2011). *Arabidopsis* ULTRAVIOLET-B-INSENSITIVE4 maintains cell division activity by temporal inhibition of the anaphase-promoting complex/cyclosome. *Plant Cell* 23, 4394-4410. 10.1105/tpc.111.091793 22167059 PMC3269873

[DEV179333C29] Holdsworth M. J., Bentsink L. and Soppe W. J. (2008). Molecular networks regulating *Arabidopsis* seed maturation, after-ripening, dormancy and germination. *New Phytol.* 179, 33-54. 10.1111/j.1469-8137.2008.02437.x 18422904

[DEV179333C30] Horvath B. M., Kourova H., Nagy S., Nemeth E., Magyar Z., Papdi C., Ahmad Z., Sanchez-Perez G. F., Perilli S., Blilou I. et al. (2017). *Arabidopsis* RETINOBLASTOMA RELATED directly regulates DNA damage responses through functions beyond cell cycle control. *EMBO J.* 36, 1261-1278. 10.15252/embj.201694561 28320736 PMC5412863

[DEV179333C31] Hou A., Liu K., Catawatcharakul N., Tang X., Nguyen V., Keller W. A., Tsang E. W. and Cui Y. (2005). Two naturally occurring deletion mutants of 12S seed storage proteins in *Arabidopsis thaliana*. *Planta* 222, 512-520. 10.1007/s00425-005-1555-z 15912356

[DEV179333C32] Jaakola L., Pirttilä A. M., Halonen M. and Hohtola A. (2001). Isolation of high quality RNA from bilberry (Vaccinium myrtillus L.) fruit. *Mol. Biotechnol.* 19, 201-203. 10.1385/MB:19:2:201 11725489

[DEV179333C33] Kobayashi K., Suzuki T., Iwata E., Nakamichi N., Chen P., Ohtani M., Ishida T., Hosoya H., Muller S., Leviczky T. et al. (2015). Transcriptional repression by MYB3R proteins regulates plant organ growth. *EMBO J.* 34, 1992-2007. 10.15252/embj.201490899 26069325 PMC4551348

[DEV179333C34] Kosugi S. and Ohashi Y. (2003). Constitutive E2F expression in tobacco plants exhibits altered cell cycle control and morphological change in a cell type-specific manner. *Plant Physiol.* 132, 2012-2022. 10.1104/pp.103.025080 12913157 PMC181286

[DEV179333C35] Kurien B. T. (2009). Affinity purification of autoantibodies from an antigen strip excised from a nitrocellulose protein blot. *Methods Mol. Biol.* 536, 201-211. 10.1007/978-1-59745-542-8_22 19378059

[DEV179333C36] Lau S., Slane D., Herud O., Kong J. and Jürgens G. (2012). Early embryogenesis in flowering plants: setting up the basic body pattern. *Annu. Rev. Plant Biol.* 63, 483-506. 10.1146/annurev-arplant-042811-105507 22224452

[DEV179333C37] López-Juez E., Dillon E., Magyar Z., Khan S., Hazeldine S., de Jager S. M., Murray J. A., Beemster G. T., Bögre L. and Shanahan H. (2008). Distinct light-initiated gene expression and cell cycle programs in the shoot apex and cotyledons of *Arabidopsis*. *Plant Cell* 20, 947-968. 10.1105/tpc.107.057075 18424613 PMC2390750

[DEV179333C38] Magyar Z., Bako L., Bogre L., Dedeoglu D., Kapros T. and Dudits D. (1993). Active Cdc2-genes and cell-cycle phase-specific Cdc2-related kinase complexes in hormone-stimulated alfalfa cells. *Plant J.* 4, 151-161. 10.1046/j.1365-313X.1993.04010151.x

[DEV179333C39] Magyar Z., Atanassova A., De Veylder L., Rombauts S. and Inzé D. (2000). Characterization of two distinct DP-related genes from *Arabidopsis thaliana*. *FEBS Lett.* 486, 79-87. 10.1016/S0014-5793(00)02238-9 11108847

[DEV179333C40] Magyar Z., De Veylder L., Atanassova A., Bakó L., Inzé D. and Bögre L. (2005). The role of the *Arabidopsis* E2FB transcription factor in regulating auxin-dependent cell division. *Plant Cell* 17, 2527-2541. 10.1105/tpc.105.033761 16055635 PMC1197432

[DEV179333C41] Magyar Z., Horváth B., Khan S., Mohammed B., Henriques R., De Veylder L., Bakó L., Scheres B. and Bögre L. (2012). *Arabidopsis* E2FA stimulates proliferation and endocycle separately through RBR-bound and RBR-free complexes. *EMBO J.* 31, 1480-1493. 10.1038/emboj.2012.13 22307083 PMC3321179

[DEV179333C42] Magyar Z., Bögre L. and Ito M. (2016). DREAMs make plant cells to cycle or to become quiescent. *Curr. Opin. Plant Biol.* 34, 100-106. 10.1016/j.pbi.2016.10.002 27816815

[DEV179333C43] Mariconti L., Pellegrini B., Cantoni R., Stevens R., Bergounioux C., Cella R. and Albani D. (2002). The E2F family of transcription factors from *Arabidopsis thaliana*. Novel and conserved components of the retinoblastoma/E2F pathway in plants. *J. Biol. Chem.* 277, 9911-9919. 10.1074/jbc.M110616200 11786543

[DEV179333C44] Morgan D. O. (2007). *The Cell Cycle: Principles of Control*. Oxford University Press.

[DEV179333C45] Nowack M. K., Harashima H., Dissmeyer N., Zhao X., Bouyer D., Weimer A. K., De Winter F., Yang F. and Schnittger A. (2012). Genetic framework of cyclin-dependent kinase function in *Arabidopsis*. *Dev. Cell* 22, 1030-1040. 10.1016/j.devcel.2012.02.015 22595674

[DEV179333C46] Ohto M.-A., Fischer R. L., Goldberg R. B., Nakamura K. and Harada J. J. (2005). Control of seed mass by APETALA2. *Proc. Natl. Acad. Sci. USA* 102, 3123-3128. 10.1073/pnas.0409858102 15708976 PMC549491

[DEV179333C68] O˝szi E., Papdi C., Mohamed B., Pettko-Sz A., Leviczky T., Molnar E., Ampudia C. G., Khan S., Lopez-Juez E., Horvath B. et al. (2019). E2FB interacts with RETINOBLASTOMA RELATED and regulates cell proliferation during leaf development. *Plant Physiol.* 10.1104/pp.19.00212 PMC6945829 31694902

[DEV179333C47] Papworth C., Bauer J. C., Braman J. and Wright D. A. (1996). QuikChange site-directed mutagenesis. *Strategies* 9, 3-4. 10.1080/08924562.1996.11000299

[DEV179333C48] Raz V., Bergervoet J. H. and Koornneef M. (2001). Sequential steps for developmental arrest in *Arabidopsis* seeds. *Development* 128, 243-252. 11124119 10.1242/dev.128.2.243

[DEV179333C49] Rowland B. D. and Bernards R. (2006). Re-evaluating cell-cycle regulation by E2Fs. *Cell* 127, 871-874. 10.1016/j.cell.2006.11.019 17129771

[DEV179333C50] Rubin S. M., Gall A. L., Zheng N. and Pavletich N. P. (2005). Structure of the Rb C-terminal domain bound to E2F1-DP1: a mechanism for phosphorylation-induced E2F release. *Cell* 123, 1093-1106. 10.1016/j.cell.2005.09.044 16360038

[DEV179333C51] Sadasivam S. and DeCaprio J. A. (2013). The DREAM complex: master coordinator of cell cycle-dependent gene expression. *Nat. Rev. Cancer* 13, 585-595. 10.1038/nrc3556 23842645 PMC3986830

[DEV179333C52] Saleh A., Alvarez-Venegas R. and Avramova Z. (2008). An efficient chromatin immunoprecipitation (ChIP) protocol for studying histone modifications in *Arabidopsis* plants. *Nat. Protoc.* 3, 1018-1025. 10.1038/nprot.2008.66 18536649

[DEV179333C53] Schneider C. A., Rasband W. S. and Eliceiri K. W. (2012). NIH Image to ImageJ: 25 years of image analysis. *Nat. Methods* 9, 671-675. 10.1038/nmeth.2089 22930834 PMC5554542

[DEV179333C54] Shimada T., Fuji K., Tamura K., Kondo M., Nishimura M. and Hara-Nishimura I. (2003). Vacuolar sorting receptor for seed storage proteins in *Arabidopsis thaliana*. *Proc. Natl. Acad. Sci. USA* 100, 16095-16100. 10.1073/pnas.2530568100 14657332 PMC307698

[DEV179333C55] Sozzani R., Maggio C., Varotto S., Canova S., Bergounioux C., Albani D. and Cella R. (2006). Interplay between *Arabidopsis* activating factors E2Fb and E2Fa in cell cycle progression and development. *Plant Physiol.* 140, 1355-1366. 10.1104/pp.106.077990 16514015 PMC1435807

[DEV179333C56] Sozzani R., Maggio C., Giordo R., Umana E., Ascencio-Ibañez J. T., Hanley-Bowdoin L., Bergounioux C., Cella R. and Albani D. (2010). The E2FD/DEL2 factor is a component of a regulatory network controlling cell proliferation and development in *Arabidopsis*. *Plant Mol. Biol.* 72, 381-395. 10.1007/s11103-009-9577-8 19937368

[DEV179333C57] Sun X. D., Shantharaj D., Kang X. J. and Ni M. (2010). Transcriptional and hormonal signaling control of *Arabidopsis* seed development. *Curr. Opin. Plant Biol.* 13, 611-620. 10.1016/j.pbi.2010.08.009 20875768

[DEV179333C58] Van Daele I., Gonzalez N., Vercauteren I., de Smet L., Inzé D., Roldán-Ruiz I. and Vuylsteke M. (2012). A comparative study of seed yield parameters in *Arabidopsis thaliana* mutants and transgenics. *Plant Biotechnol. J.* 10, 488-500. 10.1111/j.1467-7652.2012.00687.x 22332878

[DEV179333C59] Vandepoele K., Vlieghe K., Florquin K., Hennig L., Beemster G. T., Gruissem W., Van de Peer Y., Inzé D. and De Veylder L. (2005). Genome-wide identification of potential plant E2F target genes. *Plant Physiol.* 139, 316-328. 10.1104/pp.105.066290 16126853 PMC1203381

[DEV179333C60] Venable D. L. (1992). Size-number trade-offs and the variation of seed size with plant resource status. *Am. Nat.* 140, 287-304. 10.1086/285413

[DEV179333C61] Vicente-Carbajosa J. and Carbonero P. (2005). Seed maturation: developing an intrusive phase to accomplish a quiescent state. *Int. J. Dev. Biol.* 49, 645-651. 10.1387/ijdb.052046jc 16096971

[DEV179333C62] Wang S., Gu Y., Zebell S. G., Anderson L. K., Wang W., Mohan R. and Dong X. (2014). A noncanonical role for the CKI-RB-E2F cell-cycle signaling pathway in plant effector-triggered immunity. *Cell Host Microbe* 16, 787-794. 10.1016/j.chom.2014.10.005 25455564 PMC4282163

[DEV179333C63] Wendrich J. R. and Weijers D. (2013). The *Arabidopsis* embryo as a miniature morphogenesis model. *New Phytol.* 199, 14-25. 10.1111/nph.12267 23590679

[DEV179333C64] Winter D., Vinegar B., Nahal H., Ammar R., Wilson G. V. and Provart N. J. (2007). An “electronic fluorescent pictograph” browser for exploring and analyzing large-scale biological data sets. *PLoS ONE* 2, e718 10.1371/journal.pone.0000718 17684564 PMC1934936

[DEV179333C65] Yang C., Bratzel F., Hohmann N., Koch M., Turck F. and Calonje M. (2013). VAL- and AtBMI1-mediated H2Aub initiate the switch from embryonic to postgerminative growth in *Arabidopsis*. *Curr. Biol.* 23, 1324-1329. 10.1016/j.cub.2013.05.050 23810531

[DEV179333C66] Yao X., Yang H., Zhu Y., Xue J., Wang T., Song T., Yang Z. and Wang S. (2018). The canonical E2Fs are required for germline development in *Arabidopsis*. *Front. Plant Sci.* 9, 638 10.3389/fpls.2018.00638 29868091 PMC5962754

[DEV179333C67] Zappia M. P. and Frolov M. V. (2016). E2F function in muscle growth is necessary and sufficient for viability in Drosophila. *Nat. Commun.* 7, 10509 10.1038/ncomms10509 26823289 PMC4740182

