# Supplementary material for: E2FA and E2FB transcription factors coordinate cell proliferation with seed maturation
Source: Development. 2019 Nov 26;146(22):dev179333. doi: 10.1242/dev.179333 (PMC6899031; doi:10.1242/dev.179333)
Supplement: Supplementary information [file develop-146-179333-s1.pdf]

Table S1. List of primers and their sequences used for mutagenesis, and qRT-PCR, and in ChIP assay. Italic letters represent the E2F-binding site in the primer pairs used for mutagenesis of LEC2 and WRI1 promoters. The nucleotid position of primer pairs from distance of the ATG start codon of the open reading frame (ORF) of E2FA and E2FB as well as the size of the PCR products are indicated.

Gene name	Forward primer	Reverse primer
**Cloning and mutagenesis**		
**WRI1 (AT3G54320)**	CTCTGAAACGAATATATGATACTA	TAAACTCTGAGAAAGTTTAGATTT
**LEC2 (AT1G28300)**	TGATTTAAACTTTTCGCTTGGGCA	TTTTCCCGGAGAGAGAGAGAGAGA
**pWRI1-E2F-MUT**	CATTCCAACT*TTTCCAAC*AAAAAATTAGAG	TCTAATTTTTTG*TTGGAAAA*GTTGGAATG
**pLEC2-E2F-MUT**	CTCTCTCTCTCTCTCTC*TTGGAAAA*GCAAGTTTGTAC	TACAAACTTGC*TTTTCCAA*GAGAGAGAGAGAGAGAG
**<u>qRT-PCR</u>**		
**ORC2 (AT2G37560)**	TCCCGAATCACAACAAACTC	CCACAATAATGGAGGTTGA
**MCM3 (AT5G46280)**	TGGGCAGCACATGAGGAC	CACTTTGTTATCATCTTGCAGTTT
**CYCD3;1 (AT4G34160)**	GCAGCATAAGTTCAAGTGTGTAGC	AAACCGTAAGAGGCAGCTCTGG
**CDKB1;1 (AT3G54180)**	TCTGTTGGTTGTATCTTTGCTGA	CATTGCTGCTCAGTTGGTGT
**RBR (AT3G12280)**	CGCTTCCATTTTGGTTTTGA	TGAACAACAGCAGCAGCAAC
**E2FA (AT2G36010)**	CAACCCAGAAACTGCTATTGTT	GTCCGACTCATCATTTTCAAC
**E2FC (AT1G47870)**	TGCCGTTATGACAGTTCTTTAGGG	AGTGTTCCATCCTCAGCTTCCT
**E2FB (AT5G22220)**	GGACCGAGCGACAACAAA	AGGTGATCTCGTAGCAGTGGA
**WRI1 (AT3G54320)**	AAGTACTTGTACCTCGGCACCT	CAATCGCAGCCATGTCATA
**LEC2 (AT1G28300)**	GGTCCAATAACAAGAGCAGAATG	CAGCTCCATTTTGCTTCACA
**LEC1 (AT1G21970)**	GTTATGGTATGTTGGACCAATCC	TTCATCTTGACCCGACGAC
**FUS3 (AT3G26790)**	TGATACTCCCGAAGAAAGCC	CTATACTTGAAGGTCCAAACGTG
**ABI3 (AT3G24650)**	GGCAGGGATGGAAACCAGAAAAGA	GGCAAAACGATCCTTCCGAGGTTA
**UBC18 (AT5G42990)**	ACAGCAATGGACATATTTGTTTAGA	TGATGCAGACTGAACTCACTGTC
**CFP**	TGAAGTTCATCTGCACCA	CTTGTACAAGCTTCGTC
**Chromatin Immunoprecipitation (ChIP)**		
**WRI1prom1(-1036)**	ACTCGTGTGTCTGCTTAAATCA	AGCACGTGTCAATCCGAAAC
**WRIprom2(-243)**	GACAGCGTGGAGAGTAAAGC	GGAGGAAAGGGCTAATTGGG
**LEC2prom1(-1342)**	TCATGGTTAGAAATTTGGTGACAGT	TCGAAATCATAACCCATAGAACACT
**LEC2prom2(-110)**	CATCTGCAACATTTTGACTCGTT	CAGAGTTTGCGTTAGAAGAGGG

Table S2. Primers and their sequences used for the RT-PCR analysis. The size of the PCR products are indicated (bp means base pair). The left or right position of the primers refer to their location according to the T-DNA insertion.

Gene name	Forward primer	Reverse primer
**RT-PCR**		
**E2FA Start-*e2fa-1* left (816bp)**	ATGTCCGGTGTCGTACGATCT	TCCTCTGTTTGTCTGATTTGGTTG
**E2FA Start-*e2fa-2* left (980bp)**	ATGTCCGGTGTCGTACGATCT	GGTCAGCCGCTTCATCTGGA
**E2FB Start-*e2fb-1* left (702bp)**	ATGTCTGAAGAAGTACCTCAA	CTTTCTTGTGATTCTCTGATTTGGT
**E2FB Start-*e2fb-2* left (645bp)**	ATGTCTGAAGAAGTACCTCAA	TGTACTTCATCCTGTAGGTTAGC
**E2FA Start-*e2fa-1* right (938bp)**	ATGTCCGGTGTCGTACGATCT	CATGAGGAGCTTTGACGGCT
**E2FA Start-*e2fa-2* right (1090bp)**	ATGTCCGGTGTCGTACGATCT	TGCACCACTCCCATTTGTGT
**E2FB Start-*e2fb-1* right (970bp)**	ATGTCTGAAGAAGTACCTCAA	TTCATCAGCCTGAGGAATGTC
**E2FB N-term-*e2fb-2* right (518bp)**	TCTGCGAAGTCTAATAAGTCTGGA	GTGCCTTTACAGCTATCAGCG

Development • Supplementary information

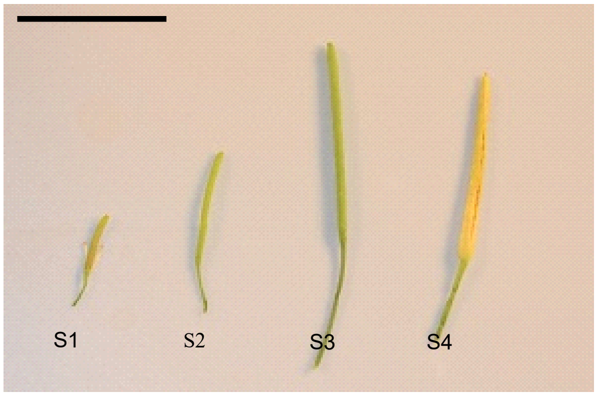

Silique stage1: 2-3 DAP
Silique stage2: 4-7 DAP
Silique stage3: 8-12 DAP
Silique stage4: 13-17 DAP

Figure S1. Silique samples were collected with different sizes representing distinct seed developmental stages. Representative pictures of the developing silique samples in four sizes; the few mm long S1, the 0,4-0,6 cm long S2, the full size siliques with green seeds (S3) and the yellow silique containing brown dry seeds referred as S4. Scale bar is 1 cm. Timing of seed development as days after pollination (DAP) is indicated on the right side.

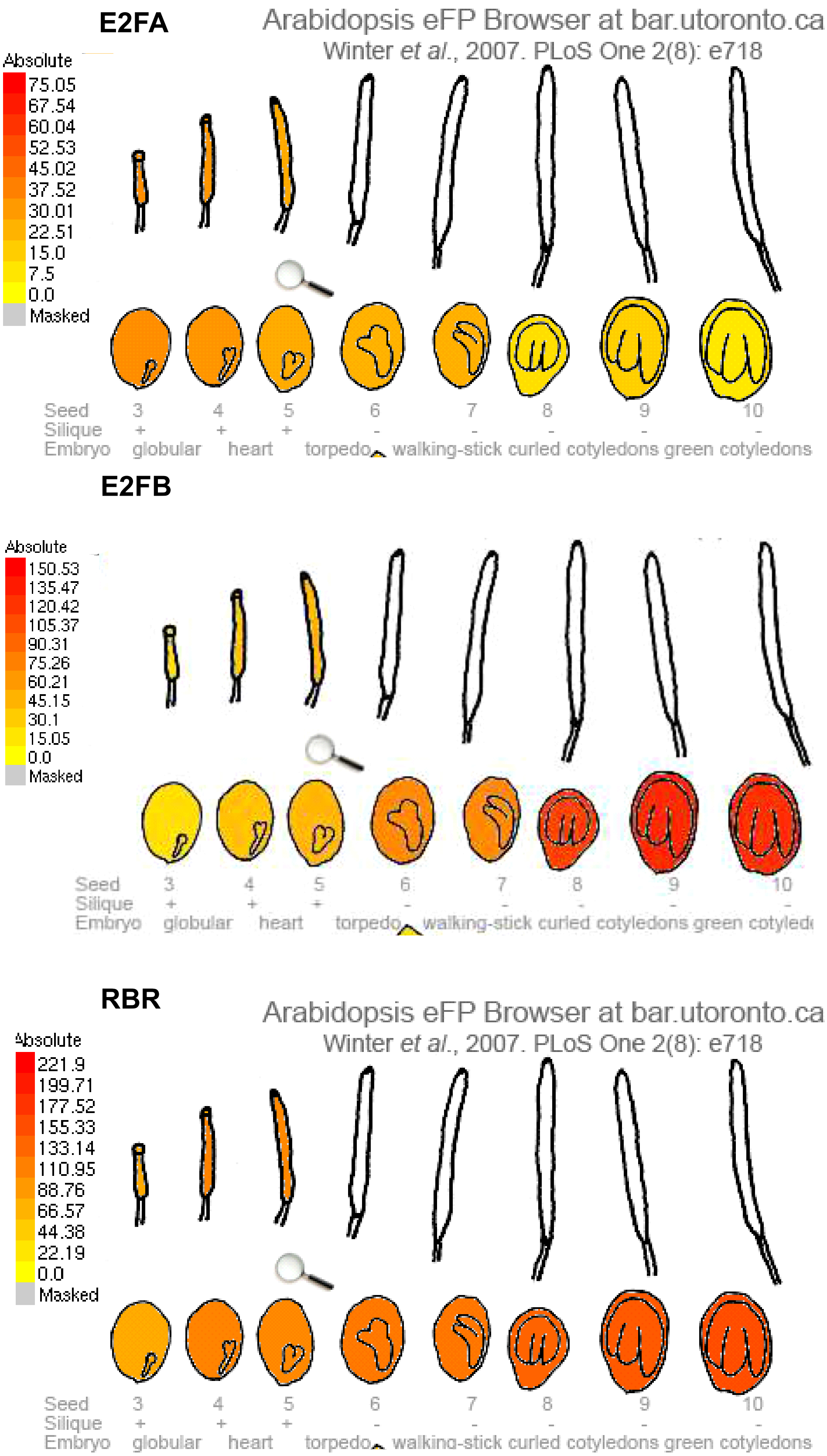

Figure S2. Seed specific expression pattern for *E2FA* (At2g36010), *E2FB* (At5g22220) and *RBR* (At3g12280) using the Arabidopsis eFP-browser (Winter et al., 2007). Values indicated correspond to Affymetrix signal values.

Development • Supplementary information

A

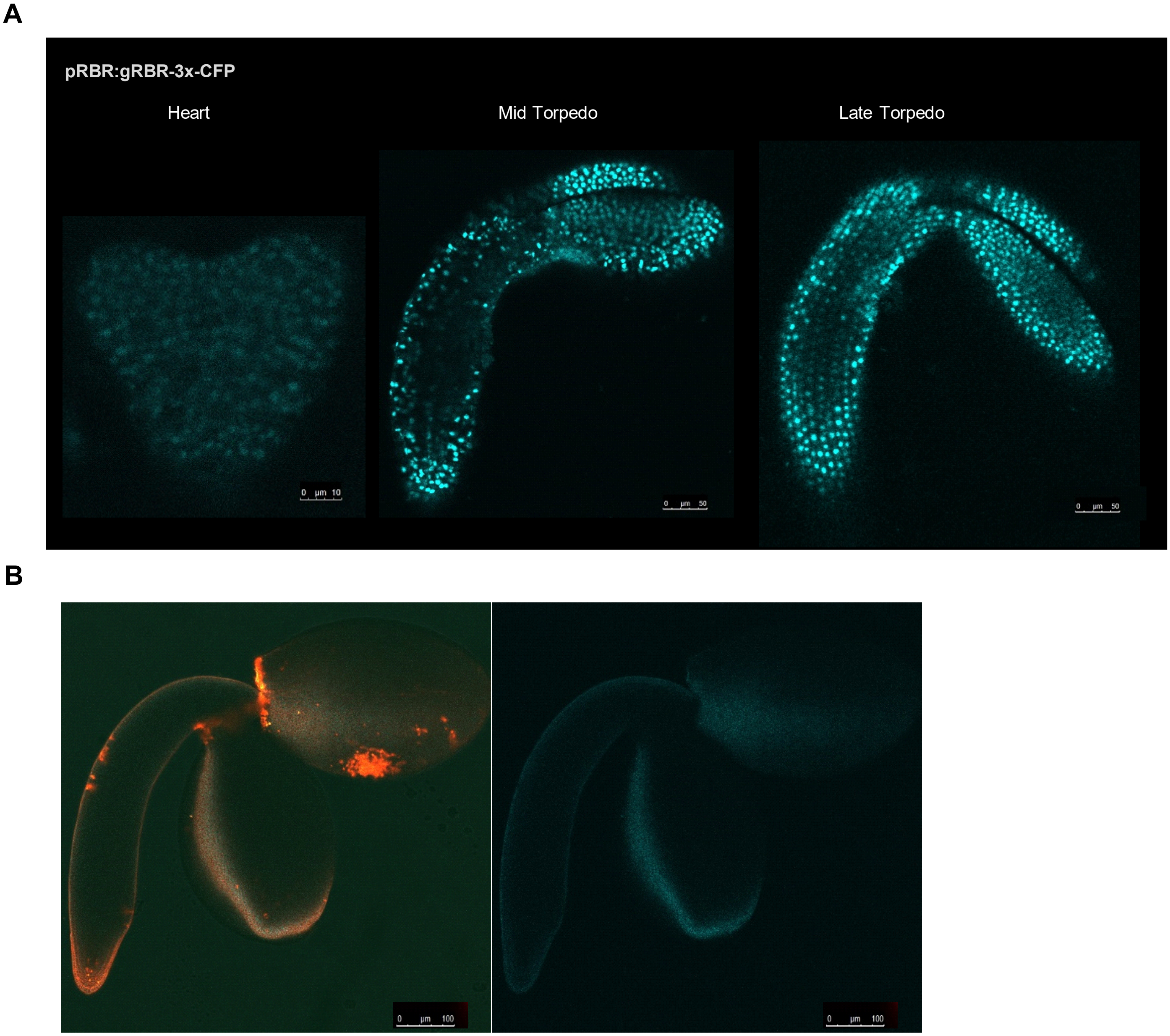

Figure S3. Accumulation of RBR protein during embryo development.

(**A**) Representative CM images of heart, late torpedo, and walking stick stage embryos of pgRBR-3xCFP line. (**B**) Representative CM image of a full-size embryo of the RBR-CFP expressing line. Red signal is propidium iodide, blue signal is CFP. Scale bars are included at the bottom side of embryo images (µm).

Development • Supplementary information

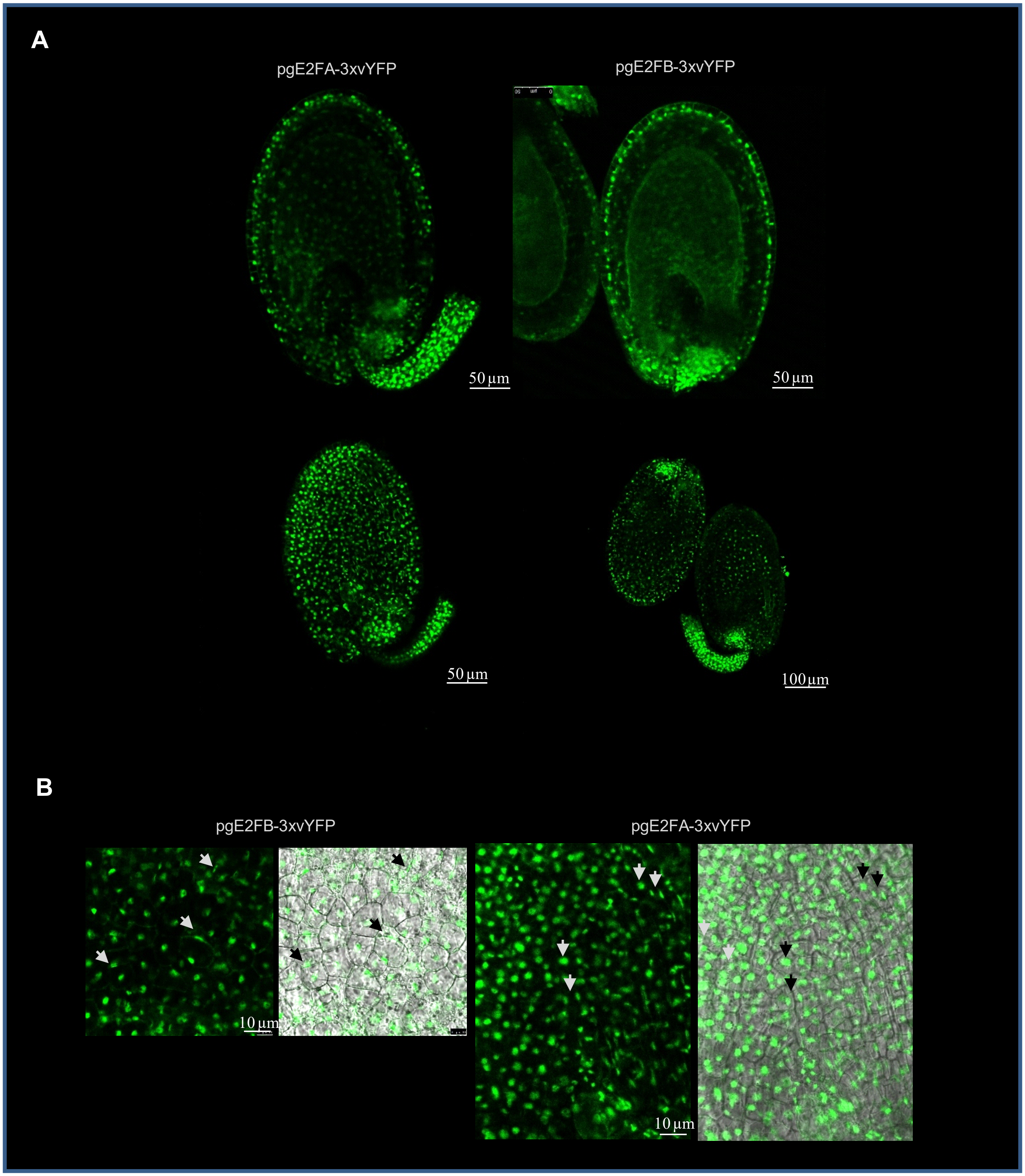

Figure S4. **Activator E2FA and E2FB proteins accumulate in the nucleus of the integument's epidermal cells.** (**A**) Representative CM images of the E2FA-GFP and E2FB-GFP expressing transgenic seeds during the morphogenic developmental phase. (**B**) Proliferating epidermal cells in the integuments of E2FA-GFP and E2FB-GFP seeds are shown by arrows. GFP signal is green. Merged GFP and bright field images are shown. Scale bars are included at the bottom side of seed images.

Development • Supplementary information

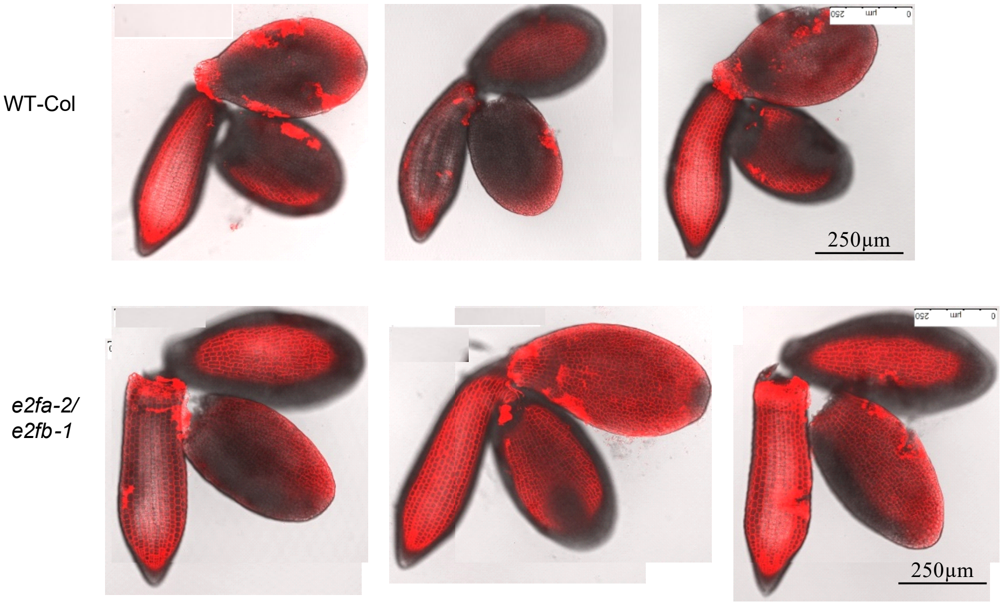

Figure S5. **The double *e2fa-2/e2fb-1* mutant embryo is larger than the control WT.** Representative CM images of propidium iodide (PI) stained embryos isolated from dry seeds of the double *e2fa-2/e2fb-1* mutants and the control WT. Merged PI-stained and bright field images. Scale bar is 250 µm.

Development • Supplementary information

WT-Col0

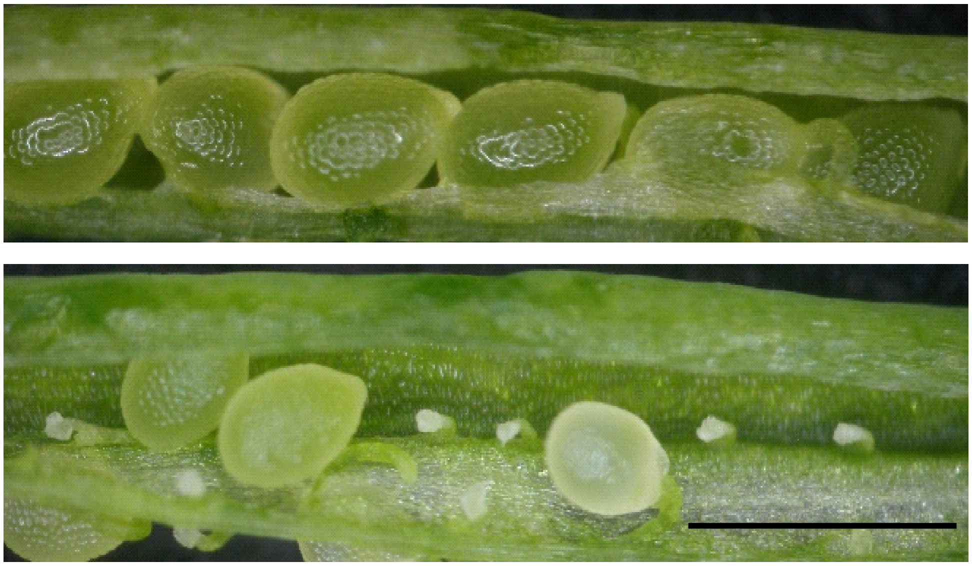

e2fa-2/e2fb-1

Figure S6. The double *e2fa-2/e2fb-1* mutant is partially sterile. Opened siliques of the WT Col control and double *e2fa-2/e2fb-1* mutant showing missing seeds in the later. Scale bar: 0,5 cm.

Development • Supplementary information

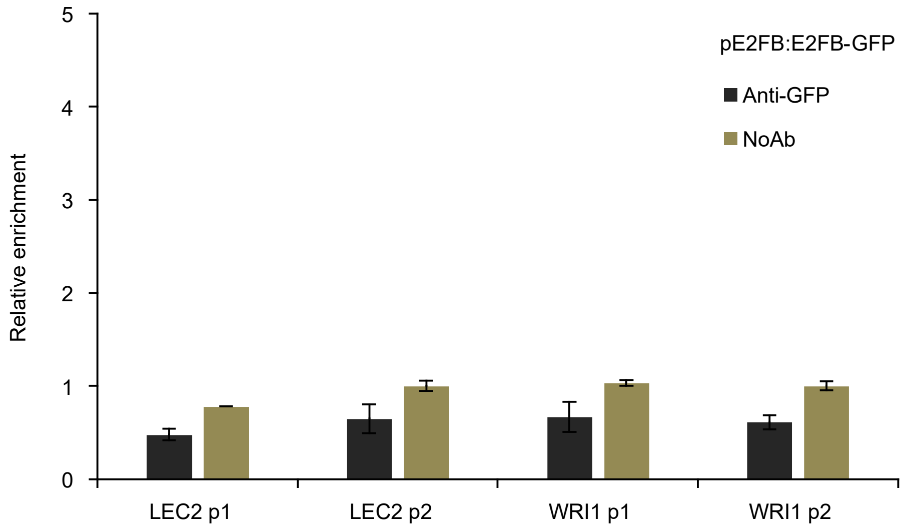

Figure S7. E2FB do not bind to the promoter region of LEC2 and WRI1 gene in maturing siliques. Chromatin immunoprecipitation (ChIP) followed by qRT-PCR using primer pairs specific for LEC2 and WRI1 promoters was carried out on chromatin isolated from developing green siliques (6-10 days after pollination - DAP) of pgE2FB-GFP transgenic line using polyclonal anti-rabbit GFP antibody. The graph shows the results of a representative experiment with three biological replicates. Nonparametric MannWhitney U test was used for statistical analysis between values of Ab and NoAb samples (* $P < 0,05$). The labels p1, p2 on x axis refer to the regions in the promoter region of LEC2 and WRI1 as indicated in Figure 6A.

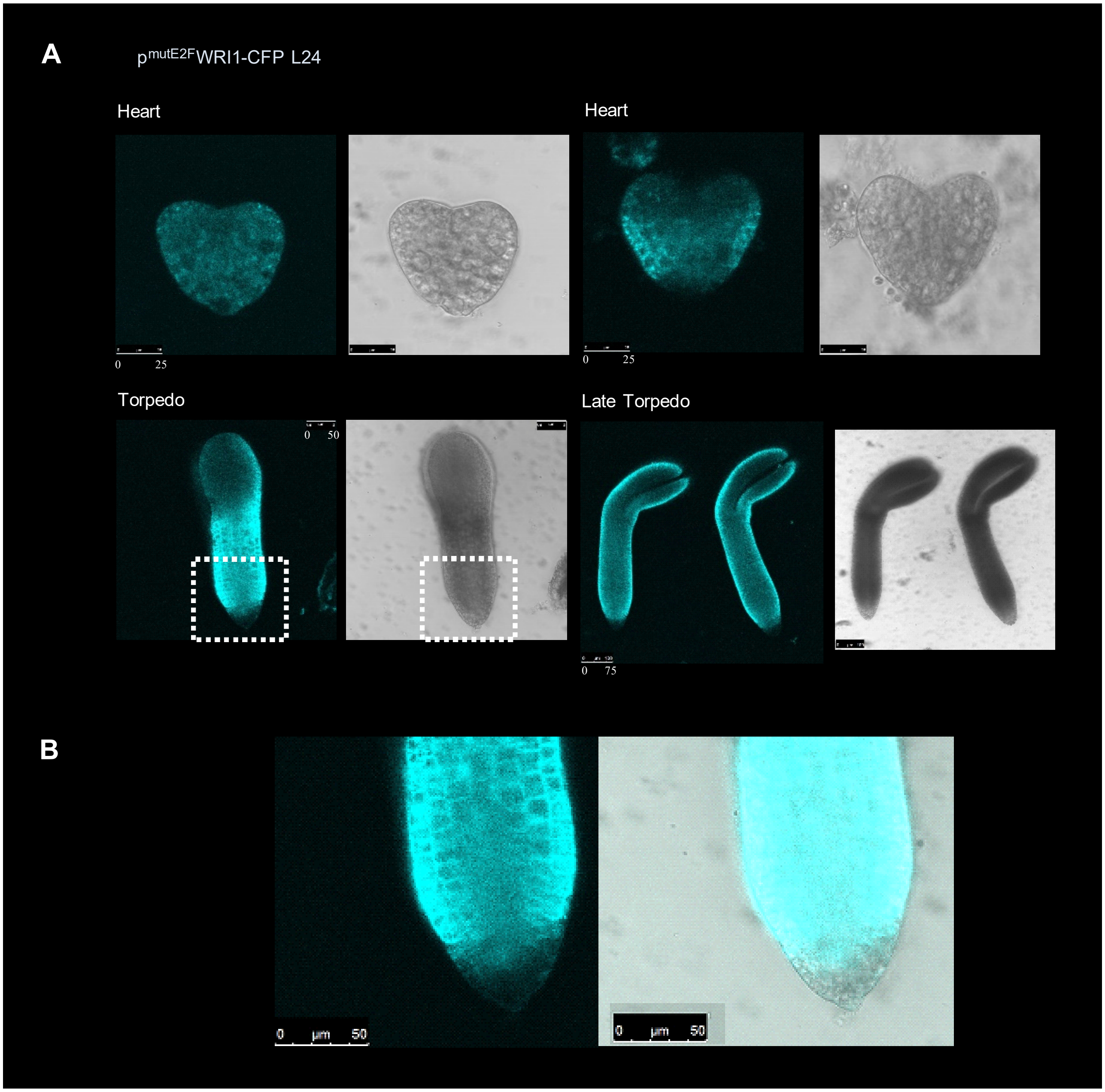

Figure S8. E2F-binding site mutant WRI1 promoter shows premature activity during embryo development. (A) Representative CM images of developing embryos from an independent E2F-binding site mutant WRI1 promoter reporter line (p^{mutE2F}WRI1::CFP L24) dissected from immature seeds. Strong CFP signal has already been detected in the heart stage embryos just like in the other E2F-binding site mutant WRI1 reporter line (p^{mutE2F}WRI1::CFP L22 - Figure 7). **(B)** The root tip regions of torpedo stage embryo outlined with open white boxes in (**A**) are enlarged in (**B**). CFP signal (blue), and a merged image with bright field are shown. Scale bars are included at the bottom side of embryo images (μm).

Development • Supplementary information

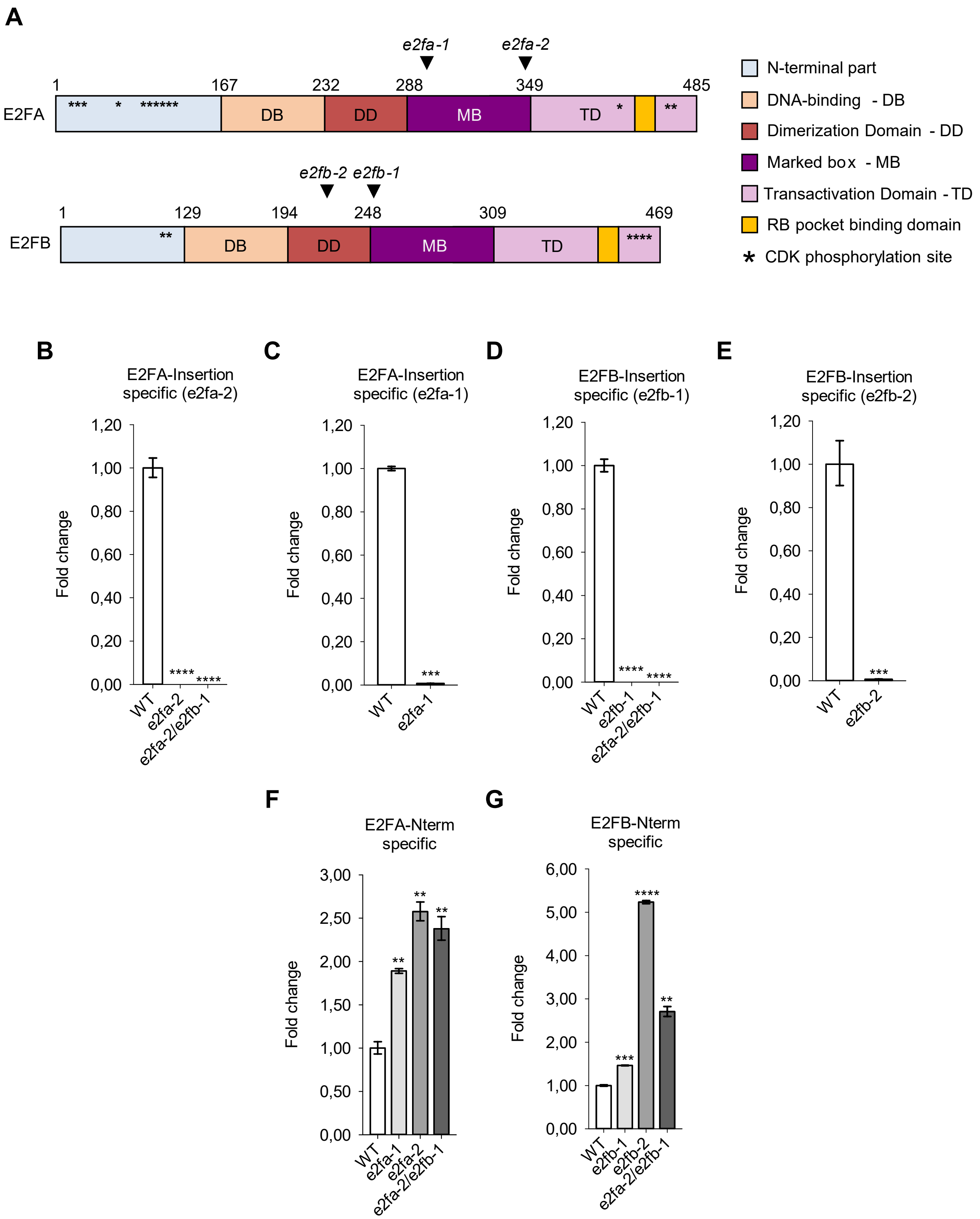

Development • Supplementary information

Figure S9. T-DNA insertion mutants for E2FA and E2FB do not express the full transcripts. (**A**) Structural organization of E2FA and E2FB proteins. Colours represent different domains as indicated. Arrowheads point to the position of the different T-DNA insertions. (**B-G**) Transcript levels of E2FA (**B, C, F**) and E2FB (**D, E, G**) in WT and T-DNA insertion mutants (*e2fa-2*, *e2fb-1*, and/or *e2fa-2/e2fb-1*). Levels of each transcript were determined by qRT-PCR using primer pairs specific for the insertion site of e2fa-2 (**B**) or *e2fa-1* (**C**), or *e2fb-1* (**D**), or *e2fb-2* (**E**), or specific for the N-terminal part of E2FA (**F**) or E2FB (**G**) preceding the insertion site. Values represent fold changes normalised to the value of the WT (set arbitrarily at 1). Data are combined from n = 3 biological repeats, and bars represent means with standard deviations. P≤0,01 (**), 0,001 (***), 0,0001 (****) were considered significant between the corresponding mutant and the WT. Abbreviations of the genes and the primer sequences are listed in Supplemental Table S1.

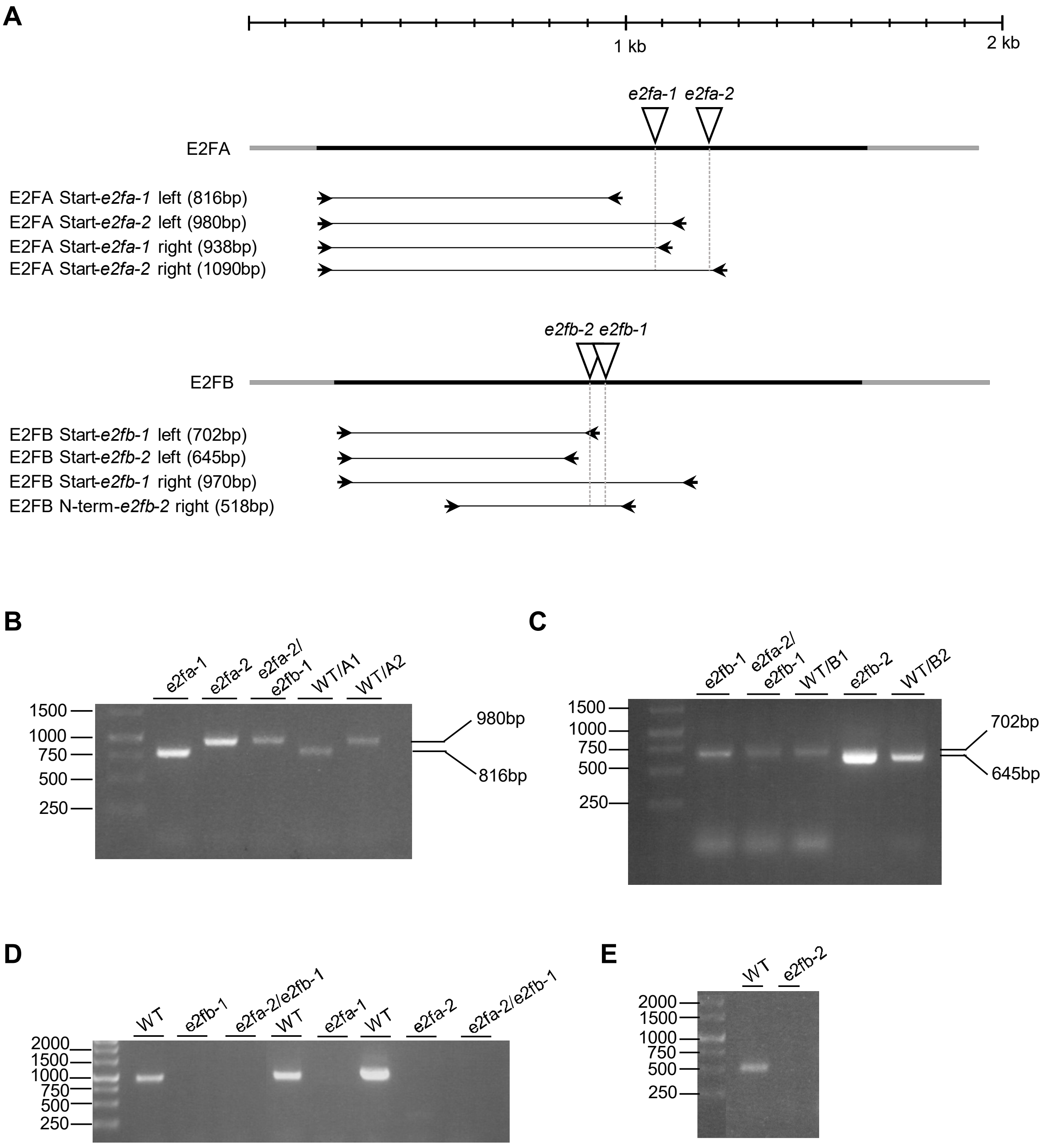

Development • Supplementary information

Figure S10. T-DNA insertion mutants for E2FA and E2FB express transcripts down to the insertion site. **(A)** Positions of the T-DNA insertions (white triangles) are shown on E2FA and E2FB mRNA, respectively. Gray and black sections indicate untranslated and translated regions. Arrows connected by black lines mark amplicons generated by specific primer pairs. Above a scale bar shows sizes in kilobases (kb). **(B-E)** cDNA prepared from WT and *e2fa* and *e2fb* single and *e2fa-2/e2fb-1* double mutant seedlings was subjected to RT-PCR analysis by using the specific primer pairs covering the entire regions in advance of the insertion (**B-C**), or beyond the insertion (**D-E**) and compared their size to the corresponding control WT products as indicated. (**B-C**) Transcripts of E2FA (**B**) and E2FB (**C**) could be transcribed in the single e2fa (*e2fa-1, e2fa-2*), e2fb (*e2fb-1 and e2fb-2*) and in the double *e2fa-2/e2fb-1* insertion mutants down to the insertion site in identical size with the control WT. (**D-E**) Transcripts were not synthesized beyond the insertion sites neither in the e2fb nor in the e2fa mutants when the reverse primers were designed next to the insertions. Primer sequences are shown in Supplemental Table S2.

Development • Supplementary information

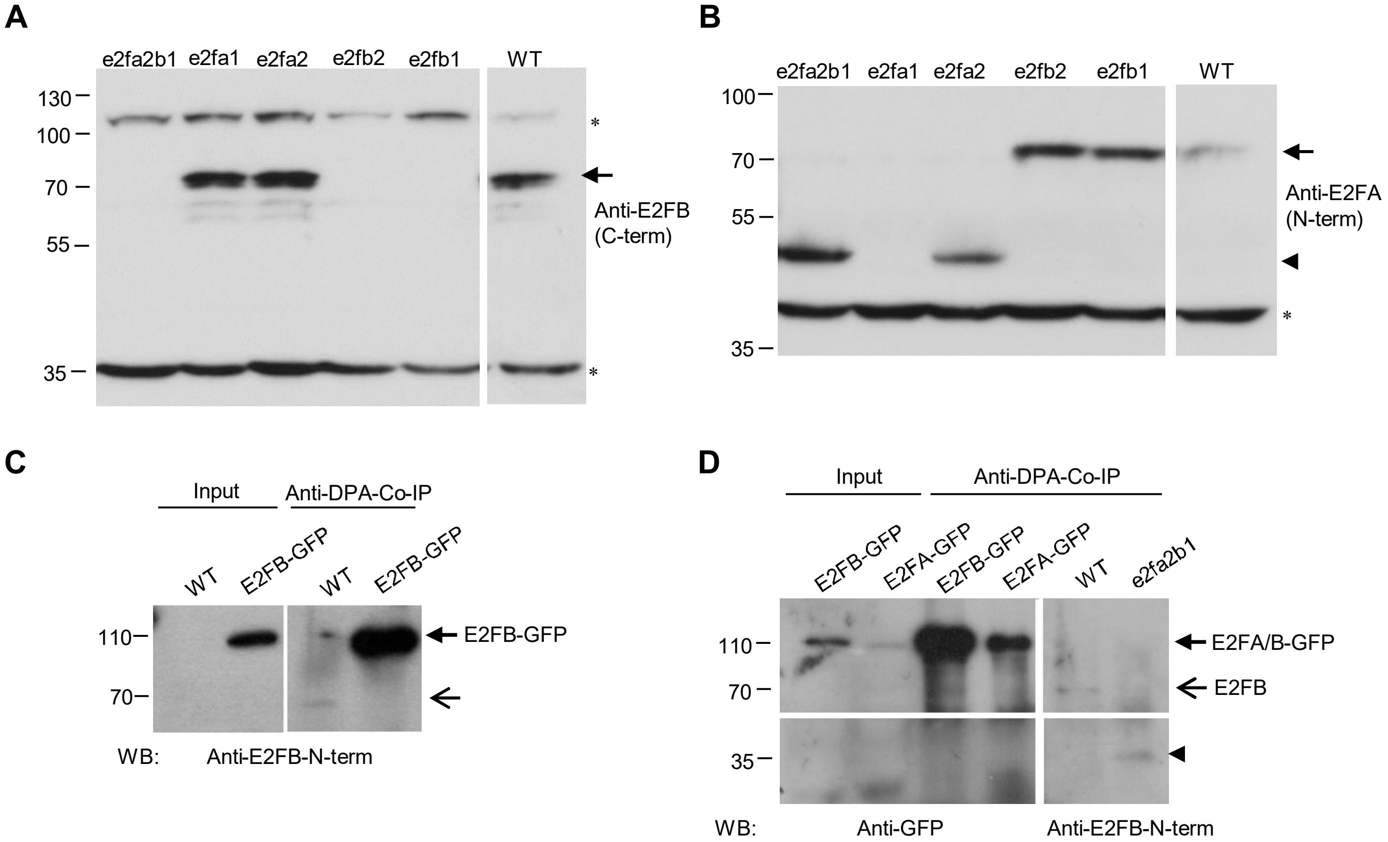

Figure S11. T-DNA insertion mutants for E2FA and E2FB synthesize proteins with smaller size than the full length E2F proteins. (**A-B**) Western blot analysis of seedlings of single and double e2fa and e2fb mutants in comparison to the WT control by using a C-terminal specific anti-E2FB (**A**), or an N-terminal specific anti-E2FA (**B**) antibody. Arrow indicates the full size E2FA or E2FB proteins, arrowhead shows a smaller size possible truncated E2FA protein (45-50kDa), while asteriks mark aspecific cross-reacting protein bands. (**C**) An N-terminal specific anti-E2FB antibody could recognize the full length endogenous E2FB in the WT control but only in the anti-DPA immunoprecipitated sample (open arrow). (**D**) By using the same antibody an E2FB protein with smaller size (about 35kDa) was recognized in the e2fa-2/e2fb-1 double mutant seedlings immunoprecipitated by anti-DPA antibody (arrowhead). As positive control anti-DPA antibody could readily immunoprecipitate the E2FA-GFP and the E2FB-GFP proteins. Arrow shows the GFP-tagged E2FA and E2FB, open arrow marks endogenous E2FB, and arrowhead shows the potentially truncated E2FB mutant form.

Development • Supplementary information
